# Goal-Directed Behavior and Instrumental Devaluation: A Neural System-Level Computational Model

**DOI:** 10.3389/fnbeh.2016.00181

**Published:** 2016-10-18

**Authors:** Francesco Mannella, Marco Mirolli, Gianluca Baldassarre

**Affiliations:** Laboratory of Computational Embodied Neuroscience, Institute of Cognitive Sciences and Technologies, National Research Council of ItalyRome, Italy

**Keywords:** computational system-level model based on leaky firing-rate neurons, goal-directed and habitual processes, Pavlovian processes, learning, devaluation behavioral experiments with rats, brain system based on basolateral-amygdala and nucleus-accumbens and multiple basal-ganglia thalamo cortex loops, instrumental manipulanda and cues, reward satiety and value

## Abstract

Devaluation is the key experimental paradigm used to demonstrate the presence of instrumental behaviors guided by goals in mammals. We propose a neural system-level computational model to address the question of which brain mechanisms allow the current value of rewards to control instrumental actions. The model pivots on and shows the computational soundness of the hypothesis for which the internal representation of instrumental manipulanda (e.g., levers) activate the representation of rewards (or “action-outcomes”, e.g., foods) while attributing to them a value which depends on the current internal state of the animal (e.g., satiation for some but not all foods). The model also proposes an initial hypothesis of the integrated system of key brain components supporting this process and allowing the recalled outcomes to bias action selection: (a) the sub-system formed by the basolateral amygdala and insular cortex acquiring the manipulanda-outcomes associations and attributing the current value to the outcomes; (b) three basal ganglia-cortical loops selecting respectively goals, associative sensory representations, and actions; (c) the cortico-cortical and striato-nigro-striatal neural pathways supporting the selection, and selection learning, of actions based on habits and goals. The model reproduces and explains the results of several devaluation experiments carried out with control rats and rats with pre- and post-training lesions of the basolateral amygdala, the nucleus accumbens core, the prelimbic cortex, and the dorso-medial striatum. The results support the soundness of the hypotheses of the model and show its capacity to integrate, at the system-level, the operations of the key brain structures underlying devaluation. Based on its hypotheses and predictions, the model also represents an operational framework to support the design and analysis of new experiments on the motivational aspects of goal-directed behavior.

## 1. Introduction

The capacity to select actions on the basis of desired goals (*goal-directed behavior*) is a fundamental evolutionary acquisition of animals' adaptive flexibility. Goal-directed behavior relies on two capabilities (Balleine and Dickinson, 1998). First, the capacity to anticipate *action outcomes*, i.e., the effects produced by the execution of actions, on the basis of previously learned action-outcome contingencies. Second, the capacity to choose between different anticipated outcomes depending on their current value computed on the basis of the nature of the rewards and the animal's current motivational state. This paper focuses on the latter process, in particular on *instrumental devaluation effects* (IDE). In a typical IDE experiment, rats are first instrumentally trained in two separate sessions to press two levers to obtain two distinct rewards, for example a food pellet and a sucrose solution (“instrumental phase”). In a second phase, one of the rewards is made freely available to the rat to induce a satiation state for it (“satiation phase”). In a third crucial phase the animal is presented with the two levers together for the first time and *in extinction*, i.e., with no reward delivery (“devaluation test”). The typical result of the experiment is that the number of pressures of the lever associated with the valued food is comparatively higher than the number of pressures for the other lever (Balleine, 1992; Balleine and Dickinson, 1998).

Knowledge about the neural substrates of goal-directed behavior has significantly advanced in the last years. Particularly important for this work is evidence on the effects on IDE of brain lesions focused on specific brain structures. Among the most important ones, lesions of the basolateral amygdala (BLA) (Blundell et al., 2001; Balleine et al., 2003), the gustatory region of the insular cortex (IC) (Balleine and Dickinson, 2000; West et al., 2012), the core part of the nucleus accumbens (NAc) (Corbit et al., 2001), and the posterior regions of the dorsomedial striatum (DMS) (Yin et al., 2005), are shown to disrupt IDE (i.e., the rats tend to press the levers with the same frequency). This results are obtained both when the lesions are performed before or after the initial instrumental training phase. This suggests that those brain structures play a role both in the acquisition and in the expression of IDE. However, recent experiments indicate a more subtle involvement of BLA and IC in these processes and that they form a closely coupled circuit. In particular, BLA seems important only for updating the incentive value of outcomes during the satiation phase (West et al., 2012; Parkes and Balleine, 2013) whereas IC seems needed to store such information and make it available during the devaluation test (Parkes and Balleine, 2013). Finally, and importantly, the lesion of the prelimbic cortex (PL; an important part of prefrontal cortex—PFC—, in particular of the medial PFC—PFCm; Passingham and Wise, 2012) or of the thalamus (Th; in particular the mediodorsal Th—MD) impairs IDE only when the lesion is made before training (Corbit and Balleine, 2003; Ostlund and Balleine, 2008; Tran-Tu-Yen et al., 2009). These results indicate that the PL and MD are needed for the acquisition but not for the expression of IDE.

Notwithstanding the large number of experiments on IDE, there are still few works proposing comprehensive system-level accounts of the neural basis of IDE and its role in goal-directed behavior (e.g., see Yin et al., 2008; Balleine et al., 2009; Balleine and O'Doherty, 2010). These works have an important theoretical value but do not achieve the operational detail of computational models. Here we address this problem and in particular we focus on two key questions: (a) how does an animal recall the motivational value of outcomes in IDE experiments? (b) how does this value support the selection of goals? We also start to address the question: what is the brain system through which the selection of goals translates into the selection of suitable actions to pursue them? These questions are important as they are at the core of our understanding of goal-directed behavior, in turn playing a central role for animals endowed with flexible cognition (Balleine and Dickinson, 1998; Mannella et al., 2013).

This work contributes to answer these questions by presenting a computational model that incorporates most of the constraints from the lesion experiments on IDE mentioned above and that accounts for them in terms of the underlying system-level brain mechanisms. The main hypothesis of the model is that during the instrumental and satiation phases the system formed by BLA and IC (henceforth “BLA/IC”) associates the perception of the manipulanda (e.g., the levers) with the motivational value of the outcomes, and then during the devaluation test it transfers such value to goal representations via the BLA/IC-NAc connections (cf. the proposal of Donahoe et al., 1997, which, however, differs from our hypothesis for its stimulus-response theoretical framework, see Section 2.2.1).

The model also incorporates and operationalizes additional hypotheses related to how the selected goal leads to bias the selection of actions to perform: (a) the brain system underlying IDE and goal-directed behavior is based on three basal ganglia-cortical (BG-Ctx) loops involving ventral basal ganglia-PFC (BGv-PFC; “limbic loop,” called here “goal loop” for the focus on goal-directed behavior), dorsomedial BG-posterior parietal cortex (BGdm-PPC; “associative loop”), and dorsolateral BG-motor cortex (BGdl-MC; “motor loop”) (Yin and Knowlton, 2006; Baldassarre et al., 2013b; Fiore et al., 2014); (b) goals are stored and selected within the goal loop (Passingham and Wise, 2012), in particular involving NAc and PL, and value information is conveyed to it from BLA/IC through NAc (Mannella et al., 2013); (c) learning processes involving the BLA/IC-NAc-PL axis are guided by cortico-cortical pathways encompassing MC, PPC, and PFC, and encoding action-outcome associations (Mannella and Baldassarre, 2015); (d) goals selected by the goal loop bias action selection processes via goal-action associations encoded in both sub-cortical pathways (involving dopaminergic spirals and dorsomedial striatum—DMS; Belin et al., 2009) and cortico-cortical pathways (involving PL, PPC, and MC; Caligiore et al., 2010; Baldassarre et al., 2013a). These hypotheses derive from: (a) empirical evidence concerning the brain structures involved in IDE; (b) general neuroscientific knowledge related to such brain structures; (c) the cited modeling/theoretical works; (d) the computational constraints generated by the model during its construction and test.

The rest of the paper is organized as follows. Section 2 presents the model structure and functioning and the biological evidence supporting them. In particular, Section 2.1 expands the evidence on lesions involving IDE addressed with the model. Section 2.2 further elaborates the main hypothesis at the core of the model. Section 2.3 explains the other hypotheses incorporated by the model. Section 2.4 explains the model at a computational detailed level. Section 3 shows how the model accounts for the target experiments. In particular, Section 3.1 illustrates the simulated environment, rats, and experiments used to test the model. Section 3.2 addresses the standard devaluation experiment with two manipulanda. Section 3.2 addresses a devaluation experiment using only one manipulandum. Section 3.4 presents some predictions of the model. Finally, Section 4 discusses the results and draws the conclusions. The acronyms used in the paper and the model parameters are indicated in the Appendix.

## 2. Materials and methods: biological evidence, hypotheses, and computational details

### 2.1. Evidence from lesion experiments

The results of a large number of lesion experiments furnish strong constraints on the brain system underlying IDE, so they have been used to build the system-level architecture of the model. As argued by some researchers (e.g., Passingham and Wise, 2012), lesion studies are a primary source of information to indicate if a brain structure is actually necessary to express a certain behavioral function. Table 1 summarizes the main results of the lesion experiments considered in this work and discussed in this section. The table does not consider the lesions performed after satiation, in particular involving BLA and IC, as they are not directly addressed by the model. However, these are further discussed below. Note that also negative results on lesions, indicating that IDE persist after lesioning a certain structure, are important as they rule out a role of such structure in the behavior under study and hence restrict the range of possible explanations of it.

**Table 1:** **Summary of the lesion studies considered here**.

**Lesioned**	**Impairment of**	**Impairment of**	**References**
**structure**	**IDE: lesion**	**IDE: lesion**	
	**before learning**	**after learning**	
BLA	V	V	Blundell et al., 2001
			Balleine et al., 2003
IC	V	V	Balleine and Dickinson, 2000
			Parkes and Balleine, 2013
NAc	V	V	Corbit et al., 2001
NAs	X	X	Corbit et al., 2001
PL	V	X	Corbit and Balleine, 2003
			Tran-Tu-Yen et al., 2009
			Ostlund and Balleine, 2008
DMS	V	V	Yin et al., 2005
OFC	X	X	Ostlund and Balleine, 2007
Hip	X	X	Corbit and Balleine, 2000
BLA-PL	X	X	Coutureau et al., 2009

*“V” and “X” indicate respectively the necessity or the lack thereoff of the structure to have IDE. BLA-PL refers to a lesion study involving the disconnection of BLA and PL through the controlateral lesion technique*.

Lesions of the BLA (Blundell et al., 2001; Balleine et al., 2003), the NAc (Corbit et al., 2001), the IC (Balleine and Dickinson, 2000), or the DMS (Yin et al., 2005), performed both before or after instrumental training, impair IDE, i.e., the ability of recalling actions differentially based on the current value associated to their outcomes. The same work showing the importance of NAc (Corbit et al., 2001) also shows that nucleus accumbens shell (NAs) is not needed for IDE. Interestingly, lesions of PL (or the MD, through which PL forms loops with BGv) impair IDE only when the lesion is made before the instrumental training but not after it, thus showing that the PL is needed for the acquisition but not for the expression of IDE (Corbit and Balleine, 2003; Ostlund and Balleine, 2008; Tran-Tu-Yen et al., 2009). Instead, the lesion of the orbitofrontal cortex (OFC) does not impair IDE, even though it impairs Pavlovian outcome devaluation effects (PDE), namely it prevents the reduction of Pavlovian responses to conditioned stimuli after the devaluation of the related unconditioned stimuli (Ostlund and Balleine, 2007). Lesions of the hippocampus (Hip) before or after instrumental learning do not impair IDE, both in rats (Corbit and Balleine, 2000) and in monkeys (Chudasama et al., 2008). Using a disconnection technique involving a combined controlateral lesion of two connected brain structures, Coutureau et al. (2009) showed that disrupting the recurrent projections between BLA and PL does not impair IDE, thus demonstrating that the direct interaction between the two is not necessary for IDE.

Recent experiments indicate a complex involvement of BLA and IC in IDE and suggest that they closely interact to form an important sub-system for outcome-related incentive learning. In particular, transient inactivation of BLA during satiation has been shown to prevent IDE, whereas its inactivation after satiation leaves IDE intact (West et al., 2012). In addition, a study using a novel disconnection technique based on controlateral lesions of BLA and IC shows that the two regions form a closely coupled circuit. In particular, BLA is important for updating the incentive value of outcomes during the satiation phase, but not to exhibit IDE. Instead, IC is needed to store incentive information and make it available during the devaluation test (Parkes and Balleine, 2013). Since the interplay of BLA and IC and their specific interactions with other brain structures appear very challenging, the model presented here abstracts over their specific role in IDE and represents them as operating as a whole subsystem underlying both the acquisition and encoding of the value of outcomes.

### 2.2. The main hypothesis of the model: stimulus-stimulus Pavlovian associations of BLA/IC and the current motivational state of the animal bias goal selection

The main hypothesis of the model is that (a) the evaluation processes of the rewards involving IDE are based on the associations between the representations of external stimuli involved in the instrumental conditioning, in particular the manipulanda, and the representations of action outcomes, in particular the rewarding foods, and that (b) the value attributed to such outcomes depends on the current state of the animal. These associations rely on mechanisms pivoting on the BLA/IC subsystem.

The amygdala complex (Amg) is a core part of the appetitive and aversive motivational system in vertebrates (Balleine and Killcross, 2006; Mirolli et al., 2010). One main function it plays is to associate the representation of biological relevant stimuli from the outer world, e.g., the sight of objects (cues, levers, etc.), with information about internal body states, e.g., related to pain, thirst, hunger, satiety, and sexual excitation (Pitkänen et al., 1995; De Olmos et al., 2004). This function relies on the particular input and output connections of Amg and on the associative learning processes taking place within it.

Information on external objects and cues reach the Amg through connections with the terminal areas of the brain ventral visual pathway, such as the temporal cortex (TC) encoding objects through abstract features (Pitkänen et al., 1995; Price and Drevets, 2010). Information on internal states reach Amg through recurrent connections with mesencephalic and diencephalic nuclei, in particular the parabrachial nucleus, the nucleus of the solitary tract, and part of the hypothalamus (Hyp; in particular the ventromedial hypothalamus): these nuclei are directly involved in the primary processing of visceral and metabolic information (Pitkänen et al., 1995; Gauriau and Bernard, 2002; King, 2006a,b; Knapska et al., 2007).

The functions played by Amg rely on two kinds of associative processes (Hatfield et al., 1996; Balleine and Killcross, 2006; Mirolli et al., 2010). The first process allows stimuli (conditioned stimuli—CS) to acquire a motivational value as rewards (unconditioned stimuli—US) and relies on BLA. This process relies on the association between CS representations and US representations (this is a stimulus-stimulus CS-US association). This association allows the “transfer of the current appetitive or aversive motivational value” of the US to the CS in the sense that all reactions associated to the US (see below) can be triggered by the CS. The test of the PDE is a means to establish that these forms of associations have been established as it shows that the responses triggered by the CS are sensitive to the manipulation of the US value (Hatfield et al., 1996; Johnson et al., 2009). Below we show how these associations are very important for IDE.

The second learning process directly associates the CS to unconditioned responses (UR; these are CS-UR associations). Once formed, when a CS is perceived these associations allow Amg to directly trigger UR without the mediation of the US representation. An experiment revealing the presence of this type of association involves the lesion of the BLA within a PDE experiment. When this is done, the CS still triggers the UR even if the related US has been devalued (Hatfield et al., 1996; Blundell et al., 2003). The additional lesion of the central nucleus of Amg (CeA) abolishes this process revealing that this Amg component is necessary for the expression of this association.

The information processed by Amg and its associative learning processes allow it to trigger various responses and modulations affecting action directed toward the outer world (see Mirolli et al., 2010, for a review). In particular, Amg can trigger a number of innate responses to cope with various biologically-relevant conditions, e.g., with threatening conditions (Davis, 1992; Killcross et al., 1997; LeDoux, 1998; Shi and Davis, 1999; Medina et al., 2002; Rosen, 2004), to drive the recall of episodic memories (Phelps, 2004; LaBar and Cabeza, 2006), and to bias the selection of goal-directed behaviors, as expanded in this work (Parkinson et al., 2000; Blundell et al., 2001; Balleine and Killcross, 2006). At the same time, the associations within Amg allow it to also trigger “responses” directed to regulate body and the overall brain functioning. In particular, Amg regulates emotional body states (e.g., the blood pressure, heart rate, energy consumption; Jolkkonen and Pitkänen, 1998; Iversen et al., 2000; Davis and Whalen, 2001) and contributes to control the production of various neuromodulators (dopamine, noradrenaline, achethylcoline, and serotonin) in turn regulating the brain overall states and learning processes (Fudge and Haber, 2000; Davis and Whalen, 2001; Fudge and Emiliano, 2003; Knapska et al., 2007).

The retrieval of the incentive value of outcomes during instrumental behavior has been shown to involve the gustatory region of the anterior insular cortex (IC; Balleine and Dickinson, 2000). In particular, in devaluation experiments bilateral lesions of IC abolish IDE with satiety outcome devaluation when assessed in extinction tests (but not if food is delivered), suggesting that the IC is critical for recalling the incentive value of outcomes during choice. The roles of BLA and IC in learning and storing information on incentive value of outcomes might be based on their strong reciprocal connections (Yamamoto et al., 1984; Augustine, 1996; Nieuwenhuys, 2012). These connections suggest the existence of a close interplay of the two structures as also shown by the direct test for which the stimulation of the BLA affects the response of IC neurons (Piette et al., 2012). The importance of BLA for learning and IC for storing information is in particular supported by evidence showing that a tetanic stimulation of BLA causes an NMDA receptor-dependent long term potentiation in the ipsilateral IC (Escobar et al., 1998; Jones et al., 1999; Escobar and Bermúdez-Rattoni, 2000). Disconnecting BLA and NAc by lesioning the BLA of one brain emisphere and the controlateral NAc abolishes IDE (Shiflett and Balleine, 2010; Parkes and Balleine, 2013) so suggesting that BLA-IC-NAc might form a three-stage circuit responsible for encoding, storing, and dispatching the value of outcomes. Indeed, the technique for disconnecting two brain structures based on the lesion of their controlateral components is equivalent to ruling out not only their direct connections but also their indirect ones, so it also eliminates the functions played by intermediate stages (e.g., IC) of the circuit starting and ending with the two targeted regions (e.g., BLA and NAc). More direct evidence on the importance of the synergistic action of the two structures comes from another devaluation experiment (Parkes et al., 2015). Here the disconnection of the two structures, performed after satiation and before the devaluation test by injecting IC with GABAA agonist muscimol and NAc with a μ-opioid receptor antagonist, again abolished IDE. Notwithstanding this evidence, the specific mechanisms through which BLA and IC specifically contribute to support their interdependent functioning and learning processes are not well understood (Parkes and Balleine, 2013). For this reason, and also for its focus on the system-level aspects of IDE, the model presented here abstracts over the specific roles of BLA and IC and considers them as a whole structure. The specific mechanisms through which the two structures play their differential functions in IDE might be addressed in a future targeted research.

The connections of BLA and IC with other structures, and the evidence of focused lesions of such structures reviewed in Section 2.1, support the idea that BLA and IC are sufficient to store the current motivational values of outcomes in IDE experiments, and to transfer it to NAc for the selection of goals. BLA (Pitkänen et al., 1995; Savander et al., 1995, 1996) and IC (Augustine, 1996; Nieuwenhuys, 2012) exchange reciprocal connections with Hip, PL, and OFC but these areas are not necessary for IDE. In particular, BLA and IC are heavily connected with the Hip via reciprocal connections and through them they support Hip learning and recall of episodic memories, in particular in relation to their emotional aspects (Pitkänen et al., 1995; Augustine, 1996; McDonald, 1998; Janes, 2015). However, Corbit and Balleine (2000) showed that lesioning the Hip does not impair IDE. Second, the interaction between Amg and IC with various areas of PFC have a great role in complex decision making processes (Bechara et al., 1999; Sterzer and Kleinschmidt, 2010; Moraga-Amaro and Stehberg, 2012). However, the lesion of PL after instrumental learning does not impair the expression of IDE (Coutureau et al., 2009). Last, several studies show that OFC, another cortical region broadly connected with BLA in a reciprocal manner, has an important role in PDE (Ostlund and Balleine, 2007) and its connections with IC are important for several cognitive processes (Augustine, 1996; Nieuwenhuys, 2012). Notwithstanding this, Ostlund and Balleine (2007) showed that OFC is not needed for the expression of IDE. Overall, this evidence shows that BLA and IC can store and retrieve information on the value of outcomes in IDE experiments without the support of those other structures.

Feedforward projections from BLA and IC to the NAc thus seem to be the main connections needed to broadcast incentive value information to downstream structures (Zahm, 2000; Voorn et al., 2004). In particular, the NAc bridges BLA/IC to ventromedial PFC (Zahm, 2000), and in this respect it represents the striatal region taking part to the basal ganglia-thalamo-cortical loops involving PL (Voorn et al., 2004). In agreement with this, Corbit et al. (2001) showed that NAc is necessary for the production of IDE. Overall, this supports the idea that NAc is the gate through which BLA/IC send outcome value information to the PFC to support goal-directed behaviors (Mannella et al., 2013). We expand this idea, important for the model, in Section 2.3.

**Figure 1:**
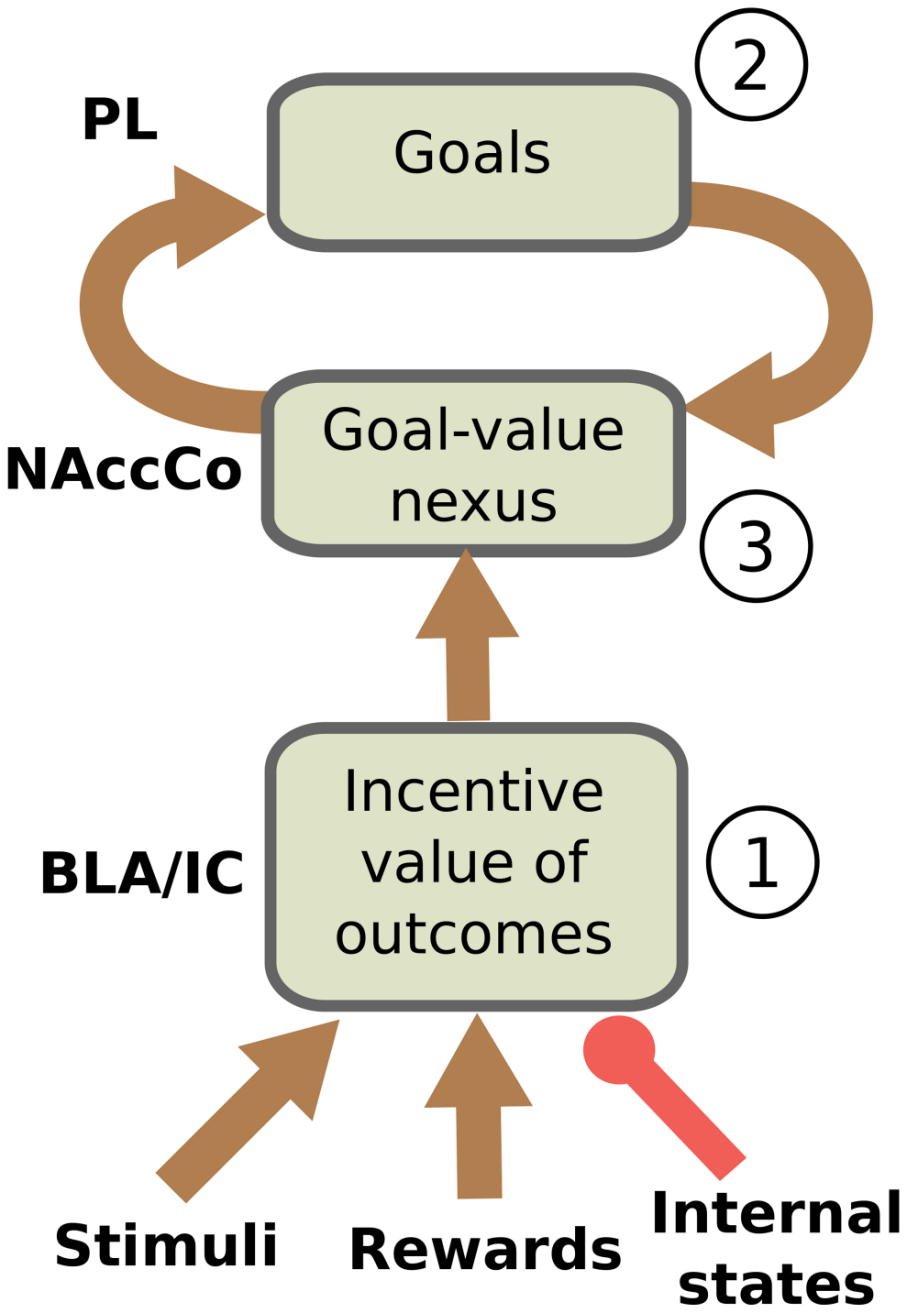
**Interactions between BLA/IC, NAc, and PL (PFC). (1)** Within BLA/IC, stimuli related to the manipulanda become associated with the stimuli related to food through Pavlovian learning processes, and at the same time the food representations are evaluated on the basis of the animal current internal states. **(2)** The PFCm, in particular PL, “proposes” the possible future outcomes that actions might cause from the current situation: the representation of these outcomes form potential goals. **(3)** The connections from BLA/IC send NAc information on the current value of possible outcomes (e.g., foods): based on this value, the NAc-PL loop selects a specific outcome to pursue (goal).

We can now restate more in detail the core hypothesis of the model based on the empirical evidence illustrated this far. The hypothesis is sketched in Figure 1. During the instrumental phase, a number of relevant learning processes take place in parallel. Instrumental learning creates the association between the sight of the context (e.g., the Skinner box) and stimuli (e.g., the lever), on one side, and actions (e.g., pressing the lever) suitable to obtain the reward (e.g., food pellets), on the other side (these associations are not in the figure). In parallel, and pivotal for the main hypothesis of the model, when instrumental learning starts to create a stimulus-reward temporal contingency also Pavlovian learning processes take off. In particular, the same sight of the stimulus (e.g., the lever) and the reward stimulus (e.g., the food pellets) that take part in instrumental learning also play the role of respectively CS and US in Pavlovian learning processes implemented in BLA/IC (Figure 1(1)). Successively, in particular in the third phase of the devaluation experiment, the associations so formed allow BLA/IC to: (a) anticipate the outcome (US; e.g., food) when the CS (e.g., lever) is perceived; (b) modulate such information based on the current animal's internal state.

As we have previously proposed (Baldassarre et al., 2013b; Mannella et al., 2013), we hypothesize that before receiving information on the value of outcomes the NAc-PL loop tends to activate the representations of the possible effects of actions that the animal could accomplish in the current context (Figure 1(2)). PFC, of which PL is part, can anticipate such effects thanks to its connections, in particular those exchanged with Hip and associative cortical areas like TC and PC (Passingham and Wise, 2012).

In the instrumental learning phase of the devaluation experiment, when BLA/IC recall the outcome representation in the presence of the CS, and at the same time the NAc-PL loop activates the representation of the possible effects of the selected actions, a third learning process can take place. This links the motivationally salient representations of outcomes in BLA/IC with the goal representations in the NAc (Figure 1(3)): the NAc thus becomes a *nexus* between incentive value information stored in BLA/IC and goal representations in PFC (Mannella et al., 2013). Later, in particular in the third phase of the devaluation experiment, this link allows the outcome value representation in BLA/IC, whose level of activation depends on the current internal state of the animal, to bias the goal-selection process taking place within the NAc-PL loop.

A further parallel learning process leads to the formation of the action-outcome/ outcome-action associations (contingencies). This learning process is studied in contingency degradation, another experimental paradigm used to operationalize goal-directed behavior alongside devaluation (Balleine and Dickinson, 1998). This learning process is not simulated in the model due our focus on IDE, so the associations it creates are assumed as already formed in the model.

#### 2.2.1. Actions are not directly recalled by Pavlovian values but via the representations of outcomes: the devaluation experiment with one manipulandum

Previous studies, most notably Donahoe et al. (1997), already proposed to interpret IDE based on the idea that manipulanda can recall the outcome incentive value. Balleine and Ostlund (2007) criticized this proposal as it tries to explain IDE within a stimulus-response (S-R) instrumental learning framework, where actions are triggered by the perception of stimuli, rather than in terms of goal-directed behavior, where actions are recalled by the anticipated re-activation of outcome representations that might be achieved with those actions. The problem of S-R interpretations is that they cannot account for the effects on behavior of the outcome value manipulations typical of IDE. It is important to clarify that the core hypothesis of the model proposed here is not an S-R hypothesis. As shown in Figure 1, the bias to select a specific action pivots on the *differential activation of goals* within the NAc-PL loop. Thus, the stimulus (e.g., the lever) does not directly recall actions, as in S-R frameworks, but rather the representation of the outcome within the BLA/IC-NAc circuit. The activation of this representation has an intensity that depends on the internal current state of the animal (e.g., hungry vs. satiated); that is, it encodes the current value of the outcomes, and this value differentially biases the selection of goals within the NAc-PL loop. The recalled outcome (O) biases the selection of the goal within the goal loop, and in turn this, and not the stimulus (S), biases the selection of actions (A) in lower BG-Ctx loops (O-A link).

To empirically rule out a possible S-R interpretation of IDE experiments, Balleine and Ostlund (2007) carried out a devaluation experiment where, in the first instrumental phase, the rats learned (in separate experimental sessions) to perform two different actions on *one* manipulandum, and these actions led, as usual, to two different outcomes. The two different actions consisted in pushing a pole either toward one direction or toward the opposite direction. As in standard devaluation experiments, the experimenters later satiated the rats for one reward, performed the devaluation test using the unique manipulandum, and measured which action was performed more frequently. The results showed that the rats performed more frequently the action associated with the valued outcome notwithstanding they were exposed to the unique ambiguous stimulus (the pole).

Balleine and Ostlund (2007) interpreted these results by proposing that stimulus-outcome pairs form whole different representations that get associated to distinct actions. If actions are associated with different outcomes, valued outcomes will bias the selection of the associated action. This explanation opens up a fundamental problem: what does activate the valued outcome representation? The model presented here posits that this activation relies on Pavlovian processes as these are potent mechanisms continuously forming S-O associations that regulate behavior in an adaptive fashion depending on the current animal's internal states. In particular, the model hypothesizes that during the instrumental phase the representation of the unique stimulus (pole) within the BLA forms Pavlovian associations with *both* outcomes. On this basis, during the test phase the stimulus (pole) tends to activate the representations of *both* outcomes. However, only the valued outcome representation can actually be activated as internal satiation inhibits the other. The association of the active outcome representation with the goal representation in the NAc-PL loop, illustrated above, will then trigger the suitable action even in the presence of the ambiguous external stimulus (the pole). This is possible because the proposed model is not an S-R model (where S would recall both actions). Rather, it is an S-O-A model where actions are triggered via the activation of the related outcomes (goals) which are “suggested” by the current environment situation (within PFC circuits) but are then “filtered” by the animal internal states attributing differential incentive values to them (through the BLA/IC-NAc-PL neural pathway).

### 2.3. Biology and main components of the model

**Figure 2:**
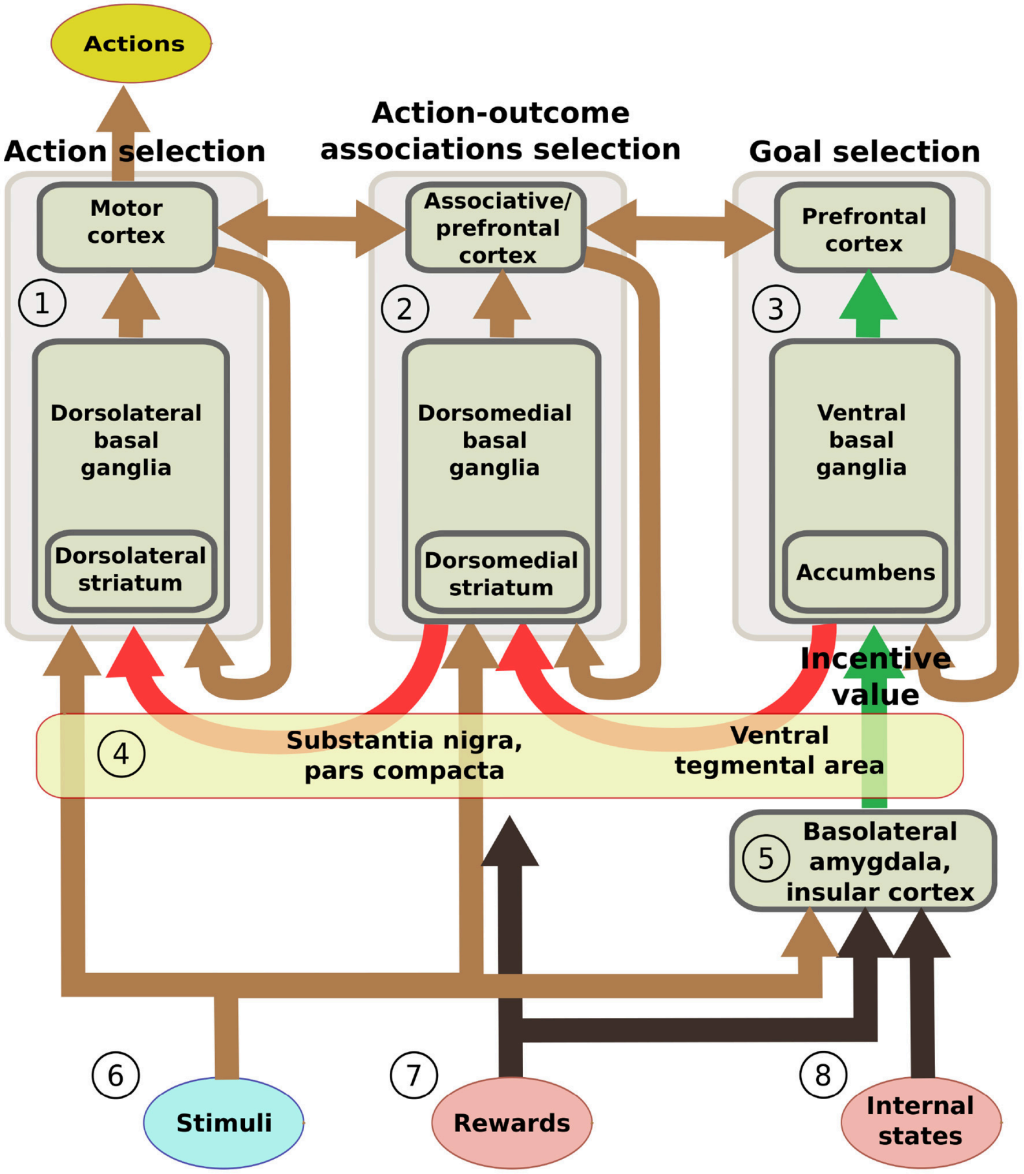
**Scheme of the main components of the model architecture**. The system is composed of three basal ganglia-thalamo-cortical loops performing respectively: **(1)** action selection (the yellow ellipse indicates the output of the system); **(2)** selection of ventral and dorsal associative cortex contents (these functions are abstracted in the model); **(3)** goal selection. BLA/IC **(5)** receive information about: **(6)** neutral stimuli (CS, e.g., the manipulanda; light blue ellipse); **(7)** rewards (US, e.g., food rewards; pink ellipse); **(8)** internal states of the animal (e.g., satiation for one food; pink ellipse). On this basis, BLA/IC elaborate the value of outcomes and communicate it to the goal loop (green arrows). Cortico-cortical projections exchange information between the goal loop, the associative loop, and the motor loop (top brown arrows). Furthermore, a parallel sub-cortical pathway relying on “dopaminergic spirals” (red arrows), formed by re-entrant connections involving different striatal regions and dopaminergic structures (VTA/SNpc), carry motivational information from the ventral to the medial and dorsal BG regions via tonic dopamine modulation **(4)**. These same dopaminergic projections also play a second role by carrying learning signals based on phasic dopamine bursts.

This section introduces five additional hypotheses that we used to structure the system-level architecture of the model within which we embed the key hypothesis presented in the previous section. While doing this, the section overviews the model architecture and functioning whereas Section 2.4 presents the model computational details. The hypotheses captures the system-level organization of key brain structures supporting the behavioral expression of IDE and involve in particular the functioning of: the BLA/IC, the striato-cortical macro-loops, the cortico-cortical connectivity, the striato-nigro-striatal pathway, and the dopamine system (Figure 2).

#### 2.3.1. The input and output information flows relevant for IDE

On the input side, the motor and associative loops and BLA/IC receive input signals from “out-of-loop” sensory cortical areas. In the model, these input cortical areas are not explicitly simulated and encode the absence/presence of the two levers with two units each activated with binary positive/zero values (Figure 2(6)). BLA/IC also receives distinct inputs, again encoded with two binary units, representing the ingestion or lack thereof of the two foods (US; Figure 2(7)). Importantly, BLA/IC also receives two input signals, again encoded with two other binary units, representing information on the food-specific satiation internal states of the animal (Figure 2(8)). VTA and SNpc receive information about the rewards as an indistinct signal (ingestion of any type of food) with the mediation of respectively the Hyp and peduncolopontine nucleus (PPN) each formed by one unit.

On the output side, two neural units of the motor loop cortex encode respectively two actions: “press the lever” and “pull the chain” (or “press lever 1” and “press lever 2”). An action is selected and performed at each time step where the related cortical unit is activated above a certain threshold.

#### 2.3.2. Basolateral amydgdala and insular cortex

As seen in Section 2.2, BLA is one main place in brain where neutral stimuli from the environment get associated with stimuli having an innate biological appetitive/aversive value depending on the internal state of the animal. In the model, the representations of food outcomes are activated either by the consumption of food or by neutral stimuli previously associated to them through a Pavlovian process. Previous computational system-level models have highlighted the importance of Amg and associative Hebbian learning rules to implement Pavlovian processes (Armony et al., 1997; Moren and Balkenius, 2000; Mannella et al., 2008; John et al., 2013; Carrere and Alexandre, 2015). These models represent an important theoretical starting point for the model presented here, but they have not traced the relations between Pavlovian processes and devaluation effects.

In the model (Figure 2(5)), the current value of food outcomes is encoded by the intensity of activation of their neural representation. In particular, the satiety state for a specific food inhibits its neural representation so that its activation, and hence value, is lower (it is lower both when the food representation is activated by a food consumption or by an anticipatory cue). An analogous mechanism has also been used in the model proposed by Zhang et al. (2009) to represent how internal states can “modulate on the fly” (i.e., without the need of a new learning) the incentive motivation value of rewards and their predicting cues. In this case a mechanism based on multiplication, rather than inhibition as here, was used, but the functional effect is the same: satiation suppresses the incentive value of foods or cues associated to them.

In the model, learning within BLA/IC takes place at the synaptic level through a time-dependent form of plasticity (Maren, 2005) modulated by phasic dopamine signals as those that might be produced by the ingestion of food (Floresco et al., 2001; Kröner et al., 2005). These processes allow BLA/IC to associate a certain stimulus perceived at a certain time (e.g., a lever or a cue) to a reward perceived at a shortly following time (e.g., food). In the model, this learning process has been implemented through a dopamine-dependent *differential Hebbian learning* rule (Kosko, 1986) capable of strengthening the connections between CS and US units when a cue is followed by a food within a certain time window.

#### 2.3.3. Basal ganglia-thalamo-cortical loops

Basal ganglia form multiple re-entrant *neural loops* with frontal and associative cortical areas (Alexander et al., 1986). These cortical areas project to different subregions of the striatum, the main input gateway of BG; in turn, these sub-regions of the striatum project to the internal component of the globus pallidum (GPi) and substantia nigra pars reticulata (SNpr), the output gateways of BG, and these project back to the same cortical areas of origin through the Th. A remarkable topological segregation is maintained within these pathways so that multiple basal ganglia-thalamo-cortical *neural channels* can be identified within each loop (Alexander et al., 1986; Haber, 2003; Voorn et al., 2004; Romanelli et al., 2005). Within the single loop, the BG component tend to activate one (or few) channels and this selectively dishinibits a specific part of the Th which in turn activates a specific neural population within cortex that possibly encodes a specific cortical content (e.g., an action, a perceptual representation, or a goal) (Redgrave et al., 1999; Grillner et al., 2005; Mannella and Baldassarre, 2015). Striatum receives not only the “within-loop” cortical connections described above, but also “out-of loop” connections from frontal and associative cortical areas encoding stimuli and context (Alexander et al., 1986; Haber, 2003), a key feature reproduced in BG computational models supporting their acquisition of S-R connections by trial-and-error processes (Doya, 2000).

The main architecture of the model is based on three loops: the motor loop, the associative loop, and the goal loop (Haber, 2003; Yin and Knowlton, 2006). The motor loop (Figure 2(1)) involves the dorsolateral striatum (DLS) and the primary motor cortex, the premotor cortex, and the supplementary motor areas (here referred to as motor cortex as a whole, MC) (Romanelli et al., 2005; Redgrave and Gurney, 2006). This loop learns by trial-and-error to select instrumental actions based on current stimuli (S-R). In the model, the motor loop selects one of the two available actions.

The associative loop (Figure 2(2)) involves the dorsomedial striatum (DMS) and parts of the PFC (in particular the dorsal PFC), the PPC, and the TC. This loop subserves the identification of locations of stimuli in space and overt/covert attention processes (Hikosaka et al., 2000; Corbetta and Shulman, 2002; Cheatwood et al., 2003; Buschman and Miller, 2007), the selection of affordances (Jeannerod et al., 1995; Buneo and Andersen, 2006), and the selection of the representations of the perceived objects (Middleton and Strick, 1996; Seger, 2008). In the model, the cortical component of the associative loop only links the representations of the motor loop with those of the goal loop (A-O and O-A associations) but it does not perform a specific processing of information. Indeed, the model does not reproduce the actual sensorimotor interactions of the animal with the environment, so objects are represented in an abstract fashion and space is not simulated. As a consequence it was not necessary to simulate attention, affordance detection, and object recognition processes taking place within the cortical areas of the associative loop.

The goal loop (Figure 2(3)) involves NAc and the orbital and medial areas of PFC (Zahm, 2000). Among these areas, the model focuses on PL as this cortical area has been shown to play a key role in goal-directed behavior and IDE (Balleine and Dickinson, 1998). PL exchanges important connections with various components of the limbic brain (NAc, Amg, Hip), and other frontal cortex areas that inform it on context and actions (Passingham and Wise, 2012). We have previously proposed that, based on this information, neural populations of PL and other close areas of PFC might represent the possible states of the environment that could be caused by the execution of the actions in the current situation (Mannella et al., 2013). The goal loop then uses incentive value information from the BLA to select and keep active some of those pre-activated representations that can hence become the animal's goals (cf. Cardinal et al., 2002; Passingham and Wise, 2012).

In the model, the BG are implemented starting from the model proposed in Gurney et al. (2001) (the main difference is that the connections carrying signals from the within-loop cortex to the striatum, simply assumed in the original model, are actually implemented in the model proposed here: see Section 2.4 for details). The BG model implements a neural competition between different possible options encoded in the target cortex on the basis of a neural circuitry that reproduces the main features of the real BG micro-architecture.

The selective function of BG is not innate but is acquired through trial-and-error learning processes (Graybiel, 1998, 2005). These processes are guided by dopaminergic phasic bursts generated by VTA, mainly targeting NAc, and SNpc, mainly targeting DLS/DMS (Schultz, 2002; Badre and Frank, 2011). In computational neuroscience (Houk et al., 1995), trial-and-error learning processes are often reproduced through the *actor-critic reinforcement learning* model, and in particualr the TD-learning rule (Sutton and Barto, 1998), capable of reproducing the typical dynamics of phasic dopamine during learning (Schultz, 2002). In the model, BG trial-and-error learning is simulated, within all the three BG-Th-Ctx loops, through a Hebbian learning process biased by dopamine. This is a simpler version of the TD-learning rule sufficient to form the needed S-R connections in the simulated rat experiments used to test the model. Dopamine phasic signals are directly produced by the VTA/SNpc on the basis of rewarding stimuli (foods) in the ways explained below. To foster exploration processes at the basis of trial-and-error learning, noise was injected into the Th component of the three loops. This noise represents in an abstract way the multiple cortical signals received by Th from various cortical sources. As an effect of learning, within the goal loop NAc representations of possible environment states get associated with the outcome representations in BLA (Figure 2(4)); within the associative loop and motor loop, respectively the DMS and DLS get associated with the current stimuli (Figure 2(4)).

#### 2.3.4. Cortico-cortical pathways

Information on desirable outcomes processed in medial and orbital PFC (part of the goal loop) is transferred to dorsal PFC (associative loop) via connections within PFC (Yeterian et al., 2011; Passingham and Wise, 2012). Information processed within the dorsal PFC affects action control via two main cortico-cortical pathways. First, through a direct pathway, involving the supplementary motor areas and MC (Babb et al., 1984; Caligiore et al., 2010), allowing PFC goals to directly affect action selection within the motor loop. Second, via the connections from the dorsal PFC to the PPC (both part of the associative loop), and the connections from the PPC to the MC. These connections are important for attention control and hence to establish the targets of action (Fox et al., 2003; Buschman and Miller, 2007) and for the top-down goal-based selection of “affordances” involved in on-line control of manipulation actions (Cavada and Goldman-Rakic, 1989; Wise et al., 1997; Borra et al., 2008).

In the model, these cortical pathways are represented as cortico-cortical connection between the cortical areas of the goal loop and the associative loop and between the cortical areas of the associative loop and the motor loop. The associative loop is hence important to encode the action-outcome associations linking the representations of outcomes/goals within the goal loop with the representations of actions within the motor loop. In the brain, the macro-structure of cortico-cortical pathways has a strong innate basis but it also undergoes cortical plasticity (Buonomano and Merzenich, 1998). In the model, however, the cortico-cortical pathways do not learn as the focus here was on IDE and not on action-outcome contingency learning and degradation.

#### 2.3.5. Striato-nigro-striatal spirals

Section 2.1 explained how lesions to either NAc or DMS impair IDE both if they are carried out before or after the instrumental training phase, whereas lesions of PL impair IDE only if they are carried out before instrumental learning but not after it. This indicates that a brain structure targeted by NAc, and different from PL, has to carry NAc information on the selected goal to the associative and motor loops. Empirical evidence suggests a possible candidate for this function, namely the striato-nigro-striatal “dopaminergic spiral” pathway that involves re-entrant connections successively involving NAc, DMS, and DLS within BG and VTA and SNpc as dopaminergic structures (Fudge and Haber, 2000; Haber, 2003). These pathway might allow the transfer of information on the incentive value of stimuli and events encoded in NAc toward DMS and DLS and the related associative and motor loops. In this respect, Belin et al. (2009) have argued that dopaminergic spirals play a key role in the formation of *incentive habits*, i.e., strong motivational automatic biases allowing Pavlovian processes of Amg to affect DMS association processes and DLS action selection processes via the NAc and the descending striato-nigro-striatal pathway departing from it.

In the model, the striato-nigro-striatal pathway plays the role of transferring the information on the current incentive value of goals encoded in NAc to the associative and motor loops based on the dopaminergic modulation of local selective processes within the BG. The connections forming the dopaminergic spirals are hardwired: the plasticity processes likely involving these connections are not simulated for the same reasons of the lack of learning in the cortico-cortical pathways. In the model, the dopaminergic spirals contain neural channels that maintain the topology throughout their stages, thus reflecting the typical segregation of other portions of the BG and the necessity for IDE of the DMS as intermediate striatal stage (Yin et al., 2005). These channels thus connect specific goal representations in NAc to specific cell assemblies in DMS and from these to specific action representations in DLS. Importantly, however, we do not have a strong commitment on this hypothesis as in real brain alternative mechanisms might carry information on goals from NAc to the associative and motor loops. For instance, dopamine control might work *temporally* rather than *spatially* as here. In this case, when, and only when, a highly-salient stimulus is perceived, it increases the dopamine efflux and this facilitates the selection supported by DMS and DLS, thus producing behaviors similar to those exhibited by our model (Belin et al., 2009; see Mannella et al., 2009, and Fiore et al., 2014, for some models using this alternative mechanism). Alternatively, information might pass through other PFC areas not considered here (Yeterian et al., 2011; Passingham and Wise, 2012). The specific mechanism used here should hence be considered as only one possible means through which NAc biases action selection in the motor loop without the support of PL. In this respect, the system-level nature of the model presented here stresses how the route followed by this information transfer is a relevant open problem.

#### 2.3.6. Dopamine

In the literature, two main distinctive functions are ascribed to phasic and tonic production of dopamine by VTA and SNpc. Phasic—intense and short-lasting—dopamine is strongly associated to plasticity of several structures of brain. Here we focus on the role of phasic dopamine in learning processes taking place within striatum (Reynolds and Wickens, 2002; Calabresi et al., 2007; Surmeier et al., 2007; Schotanus and Chergui, 2008; Shen et al., 2008) and BLA (LaLumiere et al., 2005; Marowsky et al., 2005; Li et al., 2011). Evidence from these studies shows that phasic dopamine enhances the learning processes triggered by local neural activation events, in particular giving rise to three-element Hebbian synaptic changes where synapses between two active units are strengthened in the presence of a dopamine phasic burst. In the model, rewards (e.g., caused by food ingestion) directly excite dopaminergic units of VTA/SNpc causing phasic dopamine peaks that in turn strengthen the efficacy of connections linking active couples of neurons within NAc, DMS, DLS, and BLA.

Tonic—extracellular, slow-changing—dopamine, in particular directed to NAc, has a major role in modulating animals' active coping with challenges (Salamone et al., 2007; Fiore et al., 2015). Artificial increases of tonic dopamine level, for instance induced by amphetamine, have been shown to increase the number and vigor of actions (Taylor and Robbins, 1984; Ljungberg and Enquist, 1987; see Niv et al., 2007, for a review). The mechanism leading to the increase of dopamine baseline levels relies on the inhibition of VTA/SNpc internal inhibitory units (Floresco et al., 2003). This mechanism is also reproduced in the model and leads to a lasting enhancement of striatal activation which in turn facilitates the performance of selections by the three loops.

### 2.4. The computational architecture and functioning of the model

This section describes the model in computational detail, but before doing this it presents some general considerations on its nature and on the methodology used to build it. The integrated account of the wide empirical evidence on IDE required the construction of a *system-level* model which encompasses several brain structures and their interplay. Each of these brain structures, and their connections, implement several specific functioning mechanisms and learning processes. Modeling all these elements in detail would have led us to loose our focus on IDE. We thus adopted a system-level modeling strategy reproducing in detail only the elements that were important for our hypothesis on IDE (Gurney, 2009). This strategy led us in particular to: (a) hardwire some connections of the model instead of obtaining them with learning processes: only learning processes considered central for IDE were made explicit in the model; (b) represent only in abstract ways the functioning processes of some brain structures considered in the model.

Another problem we faced was that sometimes the reproduction of the IDE experiments required the implementation of some functions relying on brain mechanisms that are still unknown. In this case, we used tentative neural mechanisms suggested by more general neuroscience knowledge and our computational experience. This approach has the advantage of allowing: (a) the formulation of operational hypotheses on IDE integrating behavioral and lesion evidence produced by several different empirical experiments; (b) the identification of current knowledge gaps of theories on IDE, in particular in relation to the neural mechanisms underlying it, and the proposal of computational hypotheses on them; (c) the production of system-level predictions testable in future empirical experiments.

**Figure 3:**
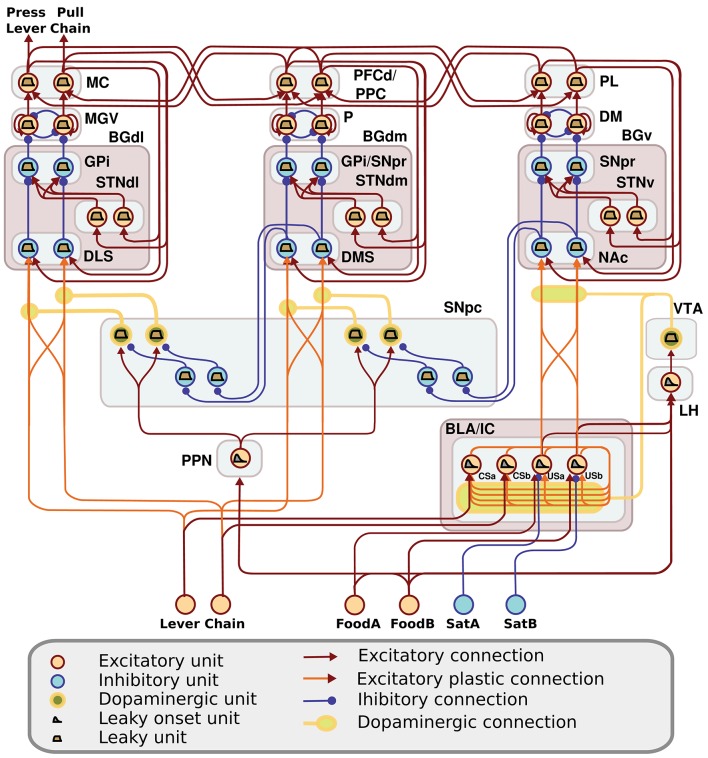
**Detailed architecture of the model, indicating its components and the neural units within them**. Learning happens at the terminals of dopaminergic connections. See Table A1 for acronyms.

The computational approach used to build the model facilitates the explanation and the reproduction of the model (the approach was initially proposed in Baldassarre et al., 2013b). In particular, the method uses uniform neural units for the whole model and few Hebbian learning rules. Thanks to this, the model can be described and understood very easily. In particular, the model can be presented through: (a) a detailed graphical scheme of its architecture which, similarly to an electrical engineering circuit, uses graphical elements to indicate the elementary components of the model (Figure 3); (b) few equations specifying the activation of the two types of neural units of the model; (c) few equations specifying the model Hebbian learning processes; (d) tables containing all the model parameters (Tables A2–A4). This information is sufficient to reproduce the model.

The detailed architecture of the model is presented in Figure 3. Overall, the architecture is based on three BG modules implemented as a modified version of the *GPR* basal ganglia model (Gurney et al., 2001). The differences between our BG module and the GPR model are as follows. First, our model does not implement the BG indirect pathway going from the striatum to the external globus pallidus (GPe), and from the GPe to the GPi and the subthalamic nucleus (STN): this pathway was not modeled as it implements regulations of the BG selection process not needed here. Second, the units of each thalamus compartment exchange reciprocal inhibitory connections thus refining the selection processes of the BG (Crabtree and Isaac, 2002; Humphries, 2002). Third, the cortical units that are targeted by a BG channel within a loop in turn project to the striatal unit of the same BG channel (this loops play an important function for guiding striatal learning based on cortical activation). Last, the STN receives afferent projections only from the cortical regions of the loop, and not from cortical areas external to it (e.g., from the input units encoding the levers), as this is suggested by anatomical evidence (Nambu, 2004; Romanelli et al., 2005). Note that the second and third hypotheses imply that the Th/Ctx initiate selections of neural representations and the BG allow them to emerge and become stable through their loops with cortex (cf. Mannella and Baldassarre, 2015). The functioning and learning processes of the model are now explained in detail.

#### 2.4.1. Input and output stages of the model

Three sets of input units are activated and reach different components of the model during simulations. Two binary units encode the absence or presence of the two manipulanda and both reach two striatal units within the motor loop, two striatal units within the associative loop, and two units within BLA/IC representing conditioned stimuli (CS). Two binary units, encoding the non-consumption or consumption of the two foods, reach two units of BLA/IC representing the unconditioned stimuli (US). The two food units also reach the single unit of the PPN, in turn activating SNpc, and the single unit of the LH, in turn activating VTA. These circuits encode the value information (reward) related to the ingestion of the two foods. Two binary units encoding no-satiation/satiation for the two foods reach, through inhibitory one-to-one connections, respectively the two BLA/IC units representing the US. The output of the model is encoded by two cortical units of the MC representing the two actions on either one of the two manipulanda. An action is triggered when the activation of one of the two units overcomes a threshold θ_*mc*_.

#### 2.4.2. The two types of neural units forming the model

The model is formed by two types of firing rate units each abstracting the activity of a whole population of neurons encoding relevant information (e.g., a lever, a food, a goal, an action). The first type of units, used in most components of the model, are *leaky units* capturing the integration in time and space of the signals reaching them, similarly to what is done by the membrane potential of neurons. (Amari, 1977; Dayan and Abbott, 2001). Formally:

(1) $$\begin{array}{ll} \tau \cdot \dot{u} & =-u+I\\ I & =\sum_{i}[w_{i}\cdot v_{i}]\;, \end{array}$$

where *u* is the activation potential of the unit, $\dot{u}$ is the rate of change (time derivative) of such potential, *I* is the sum of all signals *v*_*i*_, each multiplied by the related connection weight *w*_*i*_, sent to the unit by other units connected to it, and τ is a time constant regulating the overall speed of the unit dynamics. The activation of the leaky units is based on a positive-value saturation output function of their activation potential:

(2) $$\begin{array}{l} v={\left[tanh\left(\sigma \cdot \left(u-\theta \right)\right)\right]}^{+}\;, \end{array}$$

where *v* is the unit activation, *tanh*(*x*) is the hyperbolic function, σ is a constant defining the steepness of the hyperbolic function, θ is the unit activation threshold, and [*x*]^+^ is a function returning 0 if *x* ≤ 0 and *x* if *x* > 0.

The striatal units are leaky units as those described above but their input (and hence activation) is also enhanced by dopamine as follows:

(3) $$\begin{array}{l} \tau \cdot \dot{u}=-u+\left(\iota +\delta \cdot da\right)\cdot I\;, \end{array}$$

where τ is a time constant, ι is a parameter weighting the input to the striatum that is independent of dopamine, δ is a parameter weighting the input that is dependent on dopamine, and *da* is the activation of the dopaminergic unit projecting to the striatal unit.

The unit of PPN, the unit of LH, and the four units of BLA, are represented with a second different type of units, called here “leaky onset units,” to be able to produce fast transient responses to the input in the case of PPN and LH, or to be able to implement a learning process highly sensitive to the timing of the input signals in the case of BLA/IC. A leaky onset unit is based on two coupled leaky units, one representing an excitatory neural population processing the input signals and returning the whole output of the onset unit, and a second one representing an inhibitory neural population processing the input signals and inhibiting the first population. This complex unit produces an onset response to the input signals, namely a response that first increases and then decreases even if the input signal starts and remains high for a prolonged time. Onset units allow the production of phasic responses to the rewards, in the case of PPN and LH units, or the support of the time-sensitive learning processes of the BLA/IC illustrated below. Formally, the equations of an onset unit are as follows:

(4) $$\begin{array}{l} \begin{array}{l} \tau_{o}\cdot {\dot{u}}_{o}=-u_{o}+{\left[I-u_{i}\right]}^{+}\\ \tau_{i}\cdot {\dot{u}}_{i}\;=-u_{i}+I\;, \end{array} \end{array}$$

where *u*_*o*_ represents the first input-output population, and *u*_*i*_ represents the inhibitory population. The activation function of this type of units is the same as the one of the standard leaky units (Equation 2) applied to *u*_*o*_:

(5) $$\begin{array}{l} o={\left[tanh\left(u_{o}\right)\right]}^{+}\;, \end{array}$$

where *o* is the activation of the unit.

#### 2.4.3. Learning within the BLA/IC

BLA/IC is formed by four leaky onset units that exchange all-to-all lateral connections between them. Each connection between a pre-synaptic and a post-synaptic BLA/IC unit is updated with a Hebbian learning rule depending on the time-difference between the onset activation of the two units, and on dopamine. In particular, the learning rule is applied to a memory traces of the activation of the units: such traces allow the formation of connection weights on the basis of activations of the pre- and post-synaptic units taking place at different times (e.g., as in Pavlovian “trace conditioning” or “delay conditioning”). Traces represent slow electrochemical lasting reactions following the activation of neurons. Formally, a trace related to a unit is computed as follows:

(6) $$\begin{array}{l} \tau_{t}\cdot ṫ=-t+\alpha \cdot o\;, \end{array}$$

where *t* is the trace memory of a pre- or a post-synaptic unit, α is an amplification coefficient, and τ_*t*_ is the time constant of the trace. A connection weight between two BLA/IC units is modified on the basis of the pre- and post-synaptic traces, and dopamine, as follows:

(7) $$\begin{array}{r} \Delta w_{post,pre}=\eta_{b}\cdot {\left[da-\theta_{da,bla}\right]}^{+}\cdot {\left[{ṫ}_{post}\right]}^{+}\cdot {\left[{ṫ}_{pre}\right]}^{-}\\ \;\cdot \left(max_{w,bla}-w\right)\;, \end{array}$$

where *w*_*post, pre*_ is the connection weight and Δ its change (update), η_*b*_ is a learning coefficient, *da* is the VTA dopaminergic projection to the BLA/IC, θ_*da, bla*_ is the dopamine threshold above which dopamine allows learning to take place in the BLA/IC, *max*_*w*_ is the maximum level of the connection weight, *t* are the pre- and post-synaptic traces, ṫ indicates the time derivative of *t*, [*x*]^+^ is a function returning 0 if *x* ≤ 0 and *x* if *x* > 0, and [*x*]^−^ is a function returning −*x* if *x* ≤ 0 and 0 if *x* > 0.

This Hebbian learning rule is closely related to other types of *differential Hebbian learning* rules (Kosko, 1986; Porr and Wörgötter, 2003). The variant of the rule used here causes the connection weight to increase if there is a coincidence between the descending phase of the (memory trace of the) pre-synaptic unit activation (${\left[{ṫ}_{pre}\right]}^{-}$) and the ascending phase of the (memory trace of the) post-synaptic unit activation (${\left[{ṫ}_{post}\right]}^{+}$). In other words, the rule causes an increase of the connection weight if a pre-synaptic activation is followed by a post-synaptic activation, for example as in a CS-US sequence.

#### 2.4.4. Thalamus noise and striatal learning

The noise process driving the exploration of the system and the trial-and-error learning of striatum takes place within Th. The noise is added as input to the thalamic units and is computed on the basis of a decaying moving average as follows:

(8) $$\begin{array}{l} \begin{array}{cc} \tau \cdot ṅ & =-n+\nu z\;, \end{array} \end{array}$$

where *n* is the noise added to a Th unit, ṅ is the rate of change (time derivative) of such noise, ν is a parameter regulating the size of the noise, and *z* is a random number uniformly drawn in [−0.5, +0.5] at each simulation step.

Connections from within-loop cortical units to striatal units are not trained. These connections are however important for training the synaptic weights reaching the striatum from out-of-the-loop units as they carry information to the striatum about the cortical units of the “channels” that win the within-loop competition on the basis of the BG selection processes. For example, if a cortical unit (and the corresponding channel) encoding an action within the motor loop wins the competition and is activated, its activation is projected back to the corresponding striatal unit within the loop and this unit can associate to the unit encoding the presence of a certain lever and belonging to cortical areas outside the loop. The dopamine-dependent Hebbian learning rule used for such training is as follows:

(9) $$\begin{array}{l} \Delta w_{str,inp}=\eta_{str}\cdot {\left[da-\theta_{da,str}\right]}^{+}\cdot {\left[v_{str}-\theta_{str}\right]}^{+}\\ \cdot {\left[v_{inp}-\theta_{inp,str}\right]}^{+}\;, \end{array}$$

where *w*_*str, inp*_ is the weight of the connection linking the out-of-the-loop unit *v*_*inp*_ to the striatal unit *v*_*str*_, η_*str*_ is a learning rate characterizing striatum plasticity, *da* is the activation of the dopaminergic unit projecting to the striatal unit, and θ_*da, str*_, θ_*str*_ and θ_*inp, str*_ are the thresholds of respectively the dopamine unit, the striatal unit, and the out-of-the-loop input unit that have to be overcome for learning to take place.

#### 2.4.5. The dopamine system

The SNpc component in the model is formed by two different modules corresponding to the DMS and DLS. Each module is formed by two couples of units. Within each couple, one unit projects to the corresponding striatal unit whereas the second unit inhibits the first unit. The inhibitory unit receives an afferent inhibitory connection from the corresponding unit of the striatal structures located one level higher in the striato-nigro-striatal hierarchy. This projection can reduce the baseline activation of the inhibitory unit so that the overall output of the couple increases. The time constant of the dopaminergic inhibitory units is set to a large value so that the baseline activation of the excitatory dopaminergic unit changes very slowly, thus mimicking tonic dopamine slow changes. The excitatory dopaminergic units of the SNpc couples receive an afferent connection from the onset unit of the PPN. In this way, when the PPN unit is activated by a reward, it causes a high peak of excitation of the SNpc dopaminergic couples mimicking phasic dopamine bursts. The VTA module is at the vertex of the dopaminergic spirals and receives only an excitatory phasic input from LH.

## 3. The model account of devaluation experiments

This section first describes how we simulated the devaluation experiments. Then it presents the performance of the model in the experiment with two levers, and the neural mechanisms underlying it, both when the model is fully functioning and when it undergoes focused lesions as those investigated in the literature. Successively, it presents similar analyses for the single-manipulandum experiment. Finally, it presents some predictions of the model.

### 3.1. The simulated environment, rats, and experiment

The model was tested with simulated rats acting in a simulated environment. Although the simulated rats and environment were quite abstract, they nevertheless reproduced the circular interaction of real animals with the environment, involving repeated close-loop cycles of input, processing, output, and environment reaction (in this respect, the model is “embodied,” Mannella et al., 2010). Each interaction cycle lasted 0.05 s. During the instrumental phase, when an action corresponding to a manipulandum present in the environment was chosen and maintained for 0.5 s, the corresponding food was delivered. An action selected in the absence of the corresponding manipulandum had no effect and the related channel (BG, Th, and Ctx units) was switched off to mimic the lack of any environmental feedback (e.g., tactile and visual feedback). The receipt of foods activated the related input variables for 1.0 s. The trial ended after the reward presentation, or in any case after a timeout of 15 s. The only difference of the devaluation phase with respect to the training phase was that no reward was delivered when a manipulandum was acted on. The model units were reset to zero at the end of each trial.

All simulations consisted of two instrumental training sessions followed by two devaluation test sessions. The satiation phase, happening between training and test, was simulated by suitably setting the satiation inputs of the model in the test sessions (see below). Each instrumental session lasted 20 min and was formed by multiple trials during which both satiety input units were set to zero. In the simulations with two manipulanda, in the first training session each rat experienced the first manipulandum and related reward (first food), whereas in the second session it experienced the second manipulandum and related reward (second food). In the simulations with one manipulandum, in the first training session only the first action could lead to a reward (first food) whereas in the second session only the second action could lead to a reward (second food).

The two test sessions lasted 2 min each during which no reward was delivered. In the first test phase, both satiety variables were set to zero. In the second test phase the satiety variable related to the first food was set to zero whereas the one related to the second food was set to one (note that in simulation it was not necessary to test the opposite satiation pattern as the symmetry of conditions was guaranted by design). In the two manipulanda experiment, two levers were used in the two test phases whereas in the one manipulandum experiment only one manipulandum was used.

The simulations were replicated several times by setting a different seed of the random-number generator so as to have different learning and test histories mimicking different rats. In the model, the lesion of a structure was reproduced by permanently setting the activation of its units to a value of zero whereas the disconnection between structures was performed by permanently setting the connection weights of the neural connections linking them to zero.

### 3.2. Two-manipulanda experiment: behavior of the model and underlying neural mechanisms

The simulation with two manipulanda was performed in nine different conditions. For each condition the simulation was replicated 40 times with different random seeds (rats) each including the two training sessions and the two test sessions. The first condition involved the intact version of the model (this condition was called CONTROL). Further four conditions tested the model with the lesion of respectively BLA/IC, NAc, DMS and PL performed before the training sessions (“BLA/IC-pre,” “NAc-pre,” “DMS-pre,” and “PL-pre”). The last four conditions tested the models with respectively the lesion of BLA, NAc, DMS and PL performed after the training phase (“BLA-post,” “NAc-post,” “DMS-post,” and “PL-post”).

During each training session, the learning process was monitored by measuring the number of pressures of the available lever in 10 time-bins, averaged over the 40 simulation repetitions and the two manipulanda. For each lesion, the mean number of actions in the different bins was compared with a one-factor ANOVA to detect the presence of learning. Table 2, reporting this analysis, shows that in all conditions learning was effective and led to an increase of the average number of lever presses in the succeeding learning phases (bins).

**Table 2:** **Simulations with two manipulanda: training phase**.

	**Mean lever presses in ten time bins**										**ANOVA**	
	**1**	**2**	**3**	**4**	**5**	**6**	**7**	**8**	**9**	**10**
CONTROL	3.49	5.01	6.16	7.03	7.44	7.73	8.03	8.25	8.30	8.43	*F*_(9, 711)_ = 479.66	*p* < 0.001
BLA/IC-pre	3.79	5.21	6.44	7.06	7.40	7.74	7.81	7.93	8.11	8.20	*F*_(9, 711)_ = 377.28	*p* < 0.001
NAc-pre	3.35	4.23	4.89	5.63	6.15	6.69	7.01	7.18	7.29	7.48	*F*_(9, 711)_ = 334.68	*p* < 0.001
DMS-pre	3.36	3.83	4.14	4.54	4.64	4.80	5.05	5.21	5.33	5.55	*F*_(9, 711)_ = 48.50	*p* < 0.001
PL-pre	3.36	4.16	4.98	5.51	6.23	6.65	6.91	7.15	7.21	7.36	*F*_(9, 711)_ = 282.33	*p* < 0.001
BLA-post	3.56	4.95	6.06	6.95	7.46	7.80	8.05	8.21	8.34	8.43	*F*_(9, 711)_ = 386.81	*p* < 0.001
NAc-post	3.60	5.21	6.30	7.03	7.48	7.86	8.06	8.26	8.36	8.45	*F*_(9, 711)_ = 461.96	*p* < 0.001
DMS-post	3.48	5.00	6.11	6.99	7.50	7.71	8.01	8.24	8.34	8.53	*F*_(9, 711)_ = 53.89	*p* < 0.001
PL-post	3.61	5.11	6.23	7.04	7.44	7.84	8.08	8.30	8.25	8.48	*F*_(9, 711)_ = 442.68	*p* < 0.001

*Each row shows the number of pressures of the available lever, averaged over 40 replications of the simulations and over the two levers, in ten 2-min time intervals. CONTROL refers to a condition with the fully operative model, whereas the other conditions refer to simulations where focused lesions of different structures of the model were performed either before training (-pre) or after training (-post)*.

Table 2 also shows different levels of performance at the end of training (last bin) in correspondence to the different lesions performed before training (lesions performed after training involve a condition like the control group). To verify the statistical significance of this, we ran an ANOVA analysis with two factors, one between subjects (different lesions) and one within subjects (training bins), considering only the pre-learning conditions (four conditions plus the control). The analysis revealed a significant effect of both the lesion [*F*_(4, 395)_ = *p* < 0.001] and the training [*F*_(9, 3555)_ = *p* < 0.001] factors. Focusing on the lesion factor, *post-hoc* pairwise *t*-tests with Bonferroni correction revealed that the final performance of all pre-learning lesion conditions was reduced relative to the control group with the only exception of BLA/IC-pre (BLA/IC-pre: *p* = 0.098; NAc-pre: *p* < 0.001; PL-pre: *p* < 0.001; DMS-pre: *p* < 0.001). The other pairwise *t*-tests showed other interesting results: (a) the significance of the lower final performance in the DMS-pre lesion with respect to both the NAc-pre lesion (*p* < 0.001) and the PL-pre lesion (*p* < 0.001); (b) the non-significant difference in performance between the NAc-pre and the PL-pre conditions (*p* = 1). Notably, the first set of these results, which were not targeted during the construction of the model (they were indeed found after a suggestion of a reviewer during the review process), agree with empirical data. In particular, in line with the model prediction empirical experiments have shown that neither BLA lesion (Balleine et al., 2003) nor IC lesion (Balleine and Dickinson, 2000) impair instrumental learning. Instead, NAc lesion (Corbit et al., 2001), PL lesion (Corbit and Balleine, 2003), and DMS lesion (Yin et al., 2005) do have a detrimental effect on such learning processes. Since it was not possible to verify the predicted relations between different lesions, as these were tested in experiments using different paradigms and measures, such predictions could be tested in future empirical experiments (see Section 3.4 for further comments).

The explanation of these effects on learning of lesions with respect to controls might be that the impairment of the goal-directed systems formed by NAc, PL, and DMS deprives the system of a means to “focus” on specific inputs and actions (cf. Fiore et al., 2014). In particular, this focusing might consist in a higher/stable activation of specific MC and DLS representations caused by the top-down input received from the goal-directed system (via the cortico-cortical connections and from the striato-nigro-striatal connections) leading to more efficient learning processes within the habit system. The BLA or IC lesion, instead, does not affect instrumental training as the lack of a preference for a specific food does not allow BLA/IC to bias the selection of a specific goal/action. The further predictions presented in Section 3.4 support this interpretation.

**Figure 4:**
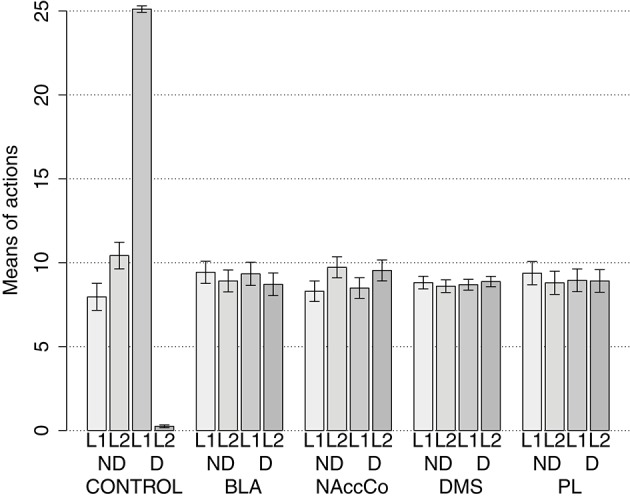
**Simulation with two manipulanda: devaluation tests, pre-training lesions**. For each condition the graph shows the number of actions performed on each manipulandum (L1 and L2), averaged over 40 replications, collected during tests without devaluation (ND) and with the devaluation of the second food (D). BLA, NAc, DMS, and PL refer to conditions where those structures were inactivated before the training phase.

IDE were measured comparing the number of actions toward one manipulandum vs. those toward the other in the two test sessions, the first with no satiation for either food and the second with satiation for the second food. IDE were considered to be in place if a statistically significant difference between the number of actions toward the two levers, measured with a *t*-test, was not present in the first test session and was present in the second test session. Table 3 and Figure 4 report the results of these tests and show that IDE were present in the CONTROL condition as in the experiments with real rats (Section 2.1).

**Table 3:** **Simulations with two manipulanda: test phase**.

	**No food devalued**				**Second food devalued**			
	**Mean actions**		***t*****-test**		**Mean actions**		***t*****-test**	
	**L1**	**L2**			**L1**	**L2**		
CONTROL	7.96	10.43	*t*_(39)_ = −1.59	*p* = 0.116	25.11	0.25	*t*_(39)_ = 91.22	*p* < 0.001
BLA/IC-pre	9.43	8.91	*t*_(39)_ = 0.39	*p* = 0.695	9.34	8.71	*t*_(39)_ = 0.46	**p** = 0.645
NAcCO-pre	8.30	9.73	*t*_(39)_ = −1.16	*p* = 0.252	8.49	9.54	*t*_(39)_ = −0.85	*p* = 0.398
DMS-pre	8.81	8.60	*t*_(39)_ = 0.28	*p* = 0.777	8.69	8.88	*t*_(39)_ = −0.30	*p* = 0.765
PL-pre	9.38	8.80	*t*_(39)_ = 0.42	*p* = 0.678	8.95	8.91	*t*_(39)_ = 0.03	*p* = 0.978
BLA-post	9.13	9.54	*t*_(39)_ = −0.28	*p* = 0.782	9.25	9.56	*t*_(39)_ = −0.22	*p* = 0.829
NAcCO-post	9.18	8.58	*t*_(39)_ = 0.41	*p* = 0.684	9.08	8.80	*t*_(39)_ = 0.18	*p* = 0.855
DMS-post	9.56	7.54	*t*_(39)_ = 1.96	*p* = 0.054	9.16	8.08	*t*_(39)_ = 1.08	*p* = 0.285
PL-post	9.61	8.56	*t*_(39)_ = 0.59	*p* = 0.554	25.09	0.35	*t*_(39)_ = 75.22	*p* < 0.001

*For each condition the means of actions on the first lever (L1) and second lever (L2), averaged over 40 replications of the simulation, are reported. The left columns refer to tests without devaluation. The right columns refer to tests where the food associated to L2 had been devalued. The CONTROL row refers to simulations with the intact model, whereas the other rows refer to simulations where various structures of the model were lesioned either before training (-pre) or after training (-post)*.

The behavior of the model can be explained as follows based on direct inspection of its functioning during training and test. The plastic connection weights linking the lever units and BLA/IC, on one side, to the striatal regions of the motor, associative, and goal loops, on the other side, were set to zero at the beginning of each simulation. The neural noise injected into the three BG-Th-Ctx loops led them to initially perform random selections. During the training phase, four different learning processes take place in the model (see Figure 2). First, within the motor loop the active DLS unit encoding the selected action in MC (recall that the action selected by MC is fed back to DLS) gets associated with the currently active input unit encoding the perceived lever when the action performance is followed by a reward (food ingestion). Second, through a similar process, the associative-loop DMS unit encoding the current selection gets associated with the current input when reward is present. Third, BLA/IC form Pavlovian associations between the currently perceived stimulus (CS: lever) and reward (US: food) when they are perceived contingently and are followed by a reward signal. Importantly, this happens only after the system acquires a successful operant behavior: this means that the instrumental learning of the motor loop creates a CS-US (manipulanda-food) contingency that can be learned by the BLA Pavlovian processes. Fourth, a learning process similar to those of DLS and DMS leads the NAc active unit encoding a possible goal, here initially activated by the Th noisy activity representing inputs from different cortical sources, to form an association with the active unit of BLA/IC representing a specific outcome (food). Through this process, the unit currently active within NAc, and hence PL, acquires a “goal sematics” and a connection with BLA/IC through which it can receive activation encoding incentive value.

During the two test devaluation phases, both levers are presented together. Based on the perception of the levers (CS), BLA/IC tend to activate the related food representations within them. In the first test phase, when the model is not satiated for any one of the two foods, this tends to lead to the selection of either one of the two actions with the same chance. Instead in the second test phase, when the second food is satiated, the BLA/IC neural representation of the latter (US) is inhibited, so only one outcome representation (first non-devalued food) can actually activate. This is the key process implementing the central hypothesis of the model: a lever, acting as CS, recalls the valued representation of food within BLA/IC, i.e., a US, and this in turn leads to select a specific goal within the goal loop. The goal representation within NAc and PL leads the system to activate, via the cortico-cortical connections and the striato-nigro-striatal dopaminergic spirals, the corresponding neural unit within the associative loop and then the motor loop, thus biasing the preferential selection of the action corresponding to the valued food.

Table 3 and Figure 4 also show the absence of IDE in the four conditions of BLA/IC-pre, NAc-pre, PL-pre, and DMS-pre where critical structures of the model were lesioned before instrumental learning. These results reflect the same lack of IDE found in animals where the same brain structures were lesioned before training (Section 2.1). The model explains these results as follows. The lesion of the BLA/IC results in a lack of bias from the motivational system to the goal loop so that the current value of the anticipated outcomes cannot influence the downstream competitions in the associative and motor loops. The lesion of NAc causes a similar effect as it interrupts the crucial nexus between the BLA/IC motivational system and the goal loop, and hence the associative and motor loops. The lesion of PL prevents the learning process linking BLA/IC and NAc, guided by PL goal activation, so that the BLA/IC cannot acquire the ability to modulate the goal-selection process within the goal loop. Finally, the lesion of DMS interrupts the propagation of incentive value from the goal loop through the dopaminergic-spirals and at the same time prevents the adequate amplification of the cortico-cortical bias from PL to associative cortical areas, so the current value of expected outcomes cannot affect the selection of actions.

**Figure 5:**
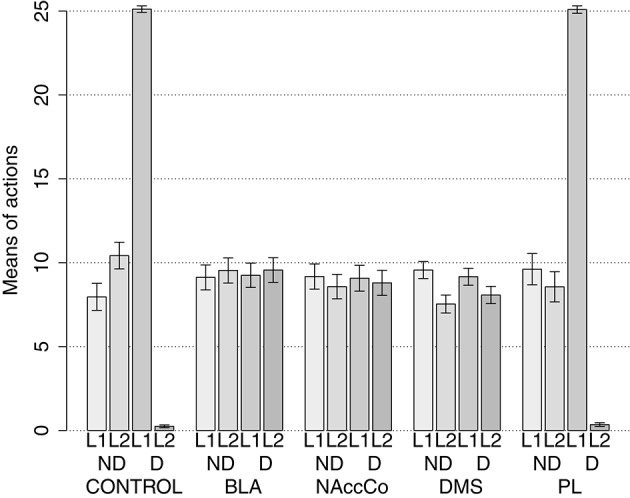
**Simulations with two manipulanda: devaluation tests, post-training lesions**. For each condition the graph shows the number of actions performed on each manipulandum (L1 and L2), averaged over 40 replications, collected during tests without devaluation (ND) and with the devaluation of the second food (D). BLA, NAc, DMS, and PL refer to conditions where those structures were inactivated after the training phase.

Table 3 and Figure 5 show that the BLA-post, NAc-post and DMS-post conditions lead to impair IDE whereas PL-post does not. These behaviors of the model reflect what happens in real rats (Section 2.1). The model explains these results as follows. As pre-training lesions, lesions of BLA/IC or NAc prevent the expression of a motivational bias in favor of the valued outcome within the goal loop. As a consequence, this loop cannot bias the selections performed within the associative and motor loops. The lesion of the DMS does not prevent BLA/IC and NAc to express a preference for the currently valued outcome but this motivational information cannot be transferred to the motor loop via the striato-nigro-striatal pathways and cortico-cortical pathways involving the associative loop. The lesion of the PL, instead, cannot prevent the BLA/IC-NAc-DMS striato-nigro-striatal pathway to communicate the incentive value related to the currently valuable outcome to the motor loop, so that actions can be biased in its favor. Together with the result of the PL-pre lesion condition, this captures the empirical evidence showing that PL is needed for learning but not for the expression of IDE (Section 2.1). Thus, the main role of the PL is to guide NAc learning processes by suitably connecting it to BLA/IC. Once this connection is acquired the motivational information about the valuable outcome can be conveyed to the motor loop via the sub-cortical pathway (or possibly other pathways/mechanisms, see Section 2.3) so that PL is not needed for the expression of IDE.

### 3.3. One-manipulandum experiment: behavior of the model and underlying mechanisms

The experiment with one manipulandum was conducted with the same modalities as the experiment with two levers, with the only difference that only one manipulandum was used. Table 4 reports the results of the training phase of the experiment, measured as done in the experiment with two levers. The results show that the model successfully learns the two actions of pressing the manipulandum in one particular direction to gain the related reward. This is possible because, following the original experiment with real rats, the two actions are trained in two different instrumental sessions so that the model can learn to consistently select the action that leads to the food of the related session. In particular, in the first session the model forms an association between the manipulandum and only the first action encoded in DLS since only the unit representing such action activates strongly due to the feedback returning to it from the corresponding within-channel MC unit. In the second session, the model forms an association between the manipulandum and the second action encoded in DLS as only this activates strongly due to the feedback received from its MC action unit. Note how it is important that in the second session the system does not wholly delete the S-R knowledge acquired in the first session due to extinction processes; here extinction was not simulated so this was not a problem, but in general it seems that acquisition processes should be stronger than extinction ones for the system to suitably learn (and/or that the stimulus S differs at least in part in the two sessions due to the different action performed).

**Table 4:** **Simulations with a single manipulandum: instrumental training phase**.

	**Mean lever presses in ten bins**										**ANOVA**	
	1	2	3	4	5	6	7	8	9	10
Means	3.73	5.10	6.29	6.98	7.38	7.46	7.89	8.05	7.94	7.98	*F*_(9, 711)_ = 245.70	*p* < 0.001

*Number of performances, averaged over 40 replications and over the training of the two actions, of the rewarded action during the training session divided in ten 2-min time intervals*.

**Figure 6:**
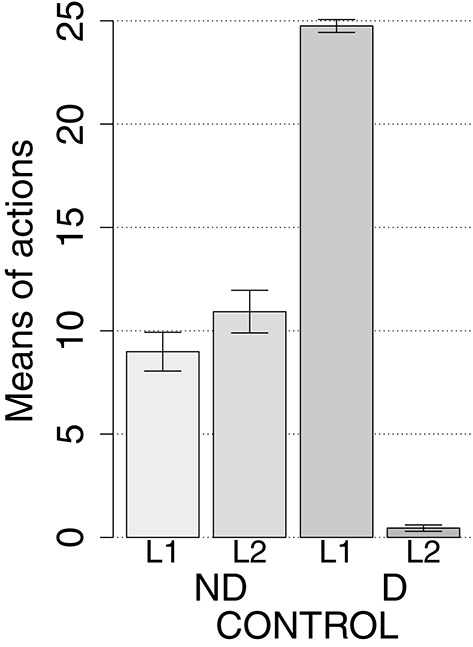
**Simulations with one manipulandum: devaluation tests**. Mean number of performances of the two actions (A1, A2) without (ND) and with devaluation of the second food (D).

The presence of IDE was measured as in the experiment with two manipulanda. Table 5 and Figure 6 show the results of the test and indicate that the model is indeed capable of successfully expressing IDE. How is this possible? In particular, how can the single manipulandum recall one or the other outcome depending on the animal internal state and lead to the correct action? The explanation is based on two elements. The first element concerns the learning process involving the BLA/IC-NAc connections during the initial instrumental phase. Within each of the two instrumental sessions, this process leads to form a connection between one BLA/IC unit, encoding the food received in that session, with one goal unit encoded in NAc. Indeed, when the food is received: (a) only one NAc unit is strongly activated by the corresponding unit of PL; (b) only one BLA/IC unit is sufficiently activated for learning as it receives the activation from both the specific ingested food and from the CS representation corresponding to the manipulandum (the CS gets associated to both food/US units). As a consequence, the US unit in BLA/IC associates only to the corresponding goal unit encoded in NAc/PL notwithstanding the unique CS encoded in BLA/IC tends to activate both food units in BLA/IC. The second element, concerning the test phase and shared with the experiment involving two levers, is related to the outcome-specific effects of satiation. The sight of the manipulandum tends to activate both US units in BLA/IC but only one food/US unit can actually activate as the other one is inhibited by the corresponding satiation variable. The active BLA/IC unit can then activate the corresponding goal unit in NAc and so bias, in downstream structures, the performance of the corresponding action.

**Table 5:** **Simulations with a single manipulandum: devaluation test phases**.

	**No food devalued**				**Second food devalued**			
	**Mean actions**		***t*****-test**		**Mean actions**		***t*****-test**	
	**A1**	**A2**			**A1**	**A2**		
Means	8.99	10.93	*t*_(39)_ = −1.01	*p* = 0.317	24.75	0.45	*t*_(39)_ = 52.64	*p* < 0.001

*Number of performances of the two actions (A1 and A2) on the manipulandum, averaged over 40 replications of the simulation, are shown. Left columns refer to tests without any devaluation. Right columns refer to tests in which the food associated to A2 has been devalued*.

### 3.4. Predictions

The data on lesions and the anatomical and physiological evidence reproduced by the model and illustrated in the previous sections represent a considerable amount of constraints satisfied by the model system-level architecture and functioning. The model can hence be used to produce predictions that might be tested in future new empirical experiments. Here we present in particular one prediction that concerns the effects of a possible lesion of the dopaminergic striato-nigro-striatal spirals that transfer incentive-value information from the goal loop to downstream loops. We have mentioned in Section 2.3 that this hypothesis of the model has been formulated in the lack of empirical evidence and that alternative hypotheses exist. For this reason it was interesting to probe the model to furnish a prediction that closely depended on that hypothesis and that would falsified the hypothesis itself if empirically disproved. To this purpose, we used the model to simulate and predict the effects of the lesion of the striato-nigro-striatal projections. In particular we set to zero the projections from the NAc to the medial region of the SNpc, and from the DMS to the dorsal region of the SNpc. The lesions were performed either before or after the instrumental training in two different simulations.

Table 6 shows that in both conditions the instrumental training was successful. However, it also shows that the lesion before training produces a comparatively slower learning and a lower steady state performance. This is due to the fact that the influence of the goal loop on the motor loop via the associative loop favors the formation of stimulus-action associations within it.

**Table 6:** **Model predictions with two manipulanda: training phase, lesions to the striato-nigro-striatal connections**.

	**Mean lever presses in ten bins**										**ANOVA**	
	**1**	**2**	**3**	**4**	**5**	**6**	**7**	**8**	**9**	**10**
CONTROL	3.49	5.01	6.16	7.03	7.44	7.73	8.03	8.25	8.30	8.43	*F*_(9, 711)_ = 479.66	*p* < 0.001
SNS-pre	3.21	4.06	4.55	4.85	5.40	5.88	6.28	6.71	6.74	7.01	*F*_(9, 711)_ = 204.31	*p* < 0.001
SNS-post	3.65	5.11	6.46	7.04	7.46	7.84	8.11	8.21	8.34	8.56	*F*_(9, 711)_ = 455.68	*p* < 0.001

*Each row reports the number of actions in ten 2-min time intervals, averaged over 40 replications of the simulation and over the two levers, in different conditions. The CONTROL row refers to simulations with the fully operative model, whereas the other two rows refer to simulations where lesions of the striato-nigro-striatal connections were performed either before (SNS-pre) or after training (SNS-post)*.

**Figure 7:**
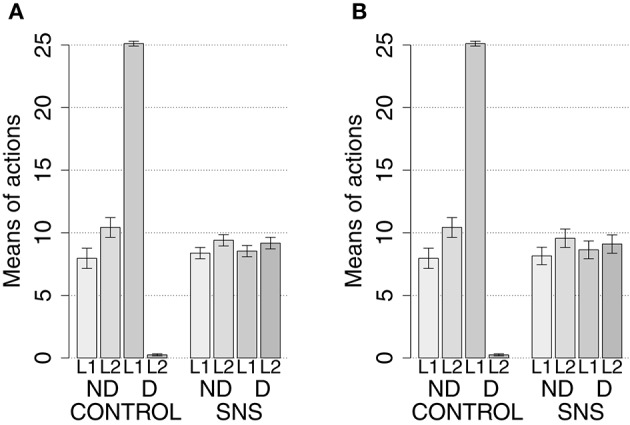
**Simulation with two manipulanda: devaluation tests, lesion of the striato-nigro-striatal connections before and after the instrumental training**. For each of the two lesions, the graphs report the number of actions on each manipulandum (L1 and L2), averaged over 40 repetitions per condition, during tests without (ND) and with devaluation (D) compared with the control condition. **(A)** Lesions to the striato-nigro-striatal connections before training. **(B)** Lesions to the striato-nigro striatal connections after training.

Table 7 and Figure 7 show the results of the devaluation tests in the two conditions. The results indicate that, in both pre- and post-training lesion conditions, the model does not express a preference for either one of the two levers, thus suggesting that the striato-nigro-striatal projections might indeed be necessary for the selected goals to affect the selection of actions. The reason of this is that the striato-nigro-striatal projections are essential to communicate incentive value information related to food from the goal loop, where this information is computed, to the motor loop, where this information is used to bias the selection of actions. In the absence of this information, the DMS leads the associative loop to perform maladaptive selections that in turn fail to suitably bias action selection within the motor loop similarly to the condition with a lesioned DMS.

**Table 7:** **Model predictions with two manipulanda: devaluation test phases, lesions of the striato-nigro-striatal connections**.

	**No food devalued**				**Second food devalued**			
	**Mean actions**		***t*****-test**		**Mean actions**		***t*****-test**	
	**L1**	**L2**			**L1**	**L2**		
CONTROL	11.88	12.05	*t*_(39)_ = −0.08	*p* = 0.937	17.13	6.43	*t*_(39)_ = 9.30	*p* < 0.001
SNS-pre	10.63	9.78	*t*_(39)_ = 1.07	*p* = 0.291	10.43	10.08	*t*_(39)_ = 0.52	*p* = 0.607
SNS-post	10.93	9.68	*t*_(39)_ = 1.32	*p* = 0.194	10.30	10.40	*t*_(39)_ = −0.15	*p* = 0.885

*Each row refers to one condition and shows the number of actions on each manipulandum averaged over 40 replications of the simulation. CONTROL refers to the condition involving the fully operative model, while the other two rows refer to the conditions where lesions of the striato-nigro-striatal pathway were performed either before (SNS-pre) or after training (SNS-post). The left columns of data refer to tests without any devaluation. The right columns of data refer to tests in which the second food was devalued*.

These results encourage the test of the model prediction in real animals. This could be done using the technique based on controlateral lesions, in this case targeting the left SNpc and the right NAc in some rats, and the right SNpc and the left NAc in others. The expectation would be that in devaluation tests IDE would be impaired although one lateral NAc component and one lateral SNpc component are still intact and can play their functions.

We close this section on predictions recalling the predictions reported in Section 3.2 and concerning the possible differential effects that different pre-instrumental-training lesions might produce on the effectiveness of learning process. As illustrated there, the model predicts that a DMS lesion would lower the final performance of rats more than either a NAc-lesion or a PL lesion; and that the latter two lesions would lead to quantitatively similar detrimental effects on learning. These predictions might be tested in future empirical experiments that use the same experimental protocol and measures to evaluate the degree to which these different lesions impair instrumental learning. Although not directly related to the devaluation paradigm, these predictions might contribute to trace how the goal-directed components of brain exert their control on action selection during the expression of IDE.

## 4. Discussion

The instrumental devaluation experimental paradigm is considered a pivotal to demonstrate the presence of goal-directed behavior in mammals (Balleine and Dickinson, 1998). The paradigm is founded on the experimental manipulation of the internal state of the animal, e.g., the satiation for a certain food but not for another one, and the demonstration that this leads to select the action directed to obtain the valued food. This shows that action selection is biased by the anticipated outcomes of actions, possibly leading to valuable resources for the animal, rather than merely by stimuli in the environment. A number of experiments with rats have identified the brain structures that are critical for these processes, in particular the basolateral amygdala, the insular cortex, the nucleus accumbens core, the dorsomedial striatum, and the prelimbic cortex (the latter necessary only for learning).

The importance of these brain structures for devaluation, the neuroscientific evidence on the pivotal role of amygdala in implementing Pavlovian processes leading to attribute value to unconditioned stimuli, and the knowledge on the role of the other brain structures in implementing goal and action selection, allowed us to propose a computational model that integrates such information in a whole operational framework. The core hypothesis of the model is that the basolateral amygdala and insular cortex re-activate the representations of action-outcomes (e.g., foods) on the basis of environment stimuli (e.g., levers) and that these representations reflect the current value of the outcomes for the animal given its current internal state (e.g., satiation for one food but not for a second one). The model also operationalizes additional hypotheses on how the incentive value computed by the amygdala and insular cortex can bias action selection by: (a) influencing goal selection performed within the basal ganglia-cortical loop involving nucleus accumbens core and some areas of the prefrontal cortex, in particular the prelimbic cortex; (b) transferring information to the downstream associative and motor basal ganglia-cortical loops via both striato-nigro-striatal dopaminergic spirals and cortico-cortical pathways.

Donahoe et al. (1997) was one of the first to propose the idea that stimuli related to manipulanda can play a key role in instrumental devaluation. However, their proposal was formulated within a stimulus-response framework and as such has been challenged by theoretical and empirical tests. Moreover, it was not further developed to account for the succeeding empirical evidence on lesions pinpointing various aspects of the brain system underlying devaluation effects. Instead, the model presented here explicitly assumes that Pavlovian processes control goal-selection cognitive processes and proposes an integrated view of how the selected goal can lead to select suitable actions via specific cortical and sub-cortical neural pathways. The model also accounts for a specific experiment specifically proposed by Balleine and Ostlund (2007) to challenge the stimulus-response account of devaluation. In this experiment rats had to use two different actions to act on one manipulandum to obtain two different food outcomes. The experiment represents a challenge for any model of devaluation that relies on external stimuli to activate outcome representations. Our model accounts for the experiment as the stimulus represented by the unique manipulandum tends to recall both outcomes but only one has incentive value based on the animal's internal state and so it can bias the selection of the related action.

An important piece of empirical evidence accounted for by the model is that the prelimbic cortex is important for the acquisition of the neural prerequisites, but not for the expression, of devaluation effects (Corbit and Balleine, 2003). This raises the problem of which brain structures transfer information on outcome value, computed by amygdala, insular cortex, and accumbens, to downstream associative and motor areas. A first possibility is that other regions of prefrontal cortex or the limbic brain, still not investigated in the literature, implement this process. These regions cannot be orbitofrontal cortex, hippocampus, and nucleus accumbens shell as these have already been ruled out by specific experiments (Corbit and Balleine, 2000; Corbit et al., 2001; Ostlund and Balleine, 2007). Here we followed the hypothesis that value information is transferred to downstream associative/motor structures via the striato-nigro-striatal pathway. This hypothesis is in line with the idea of *incentive habits*, a mechanism proposed to explain the potent effects of cues associated to the consumption of drugs of abuse, for which dopaminergic spirals support the transmission of incentive values from ventral striatum to associative and motor striatum (Belin and Everitt, 2008; Belin et al., 2009). In this respect, the model predicts that an interruption of this pathway would impair devaluation expression. Future work might test this prediction in real rats through the controlateral lesion of the nucleus accumbens core and the substantia nigra pars compacta.

Further results (noticed during the paper revision process) corroborate the soundness of the model. In particular, the model correctly predicts that the lesion of basolateral amygdala does not impair instrumental *learning* (in agreement with what empirically found in Balleine et al., 2003) whereas the lesion of the nucleus accumbens core (in agreement with Corbit et al., 2001), of the prelimbic cortex (in agreement with Corbit and Balleine, 2003), and of the dorsomedial striatum (in agreement with Yin et al., 2005) do have a detrimental effect on such learning process. These results might be due to a focusing effect exerted by the goal-directed components on the habitual ones (Fiore et al., 2014), as also suggested by some predictions produced by the model.

Related to the last point, the model also produced some predictions that might be tested in future empirical experiments. In particular, the model predicts the possible effects of a lesion of the dopaminergic striato-nigro-striatal spirals that transfer incentive-value information from the goal loop to downstream loops. In particular, the model predicts that this lesion would lead to (a) a slower learning process and performance and (b) the impairment of instrumental devaluation effects. These predictions might be tested in future work by performing a controlateral double lesion involving the right subtantia nigra pars compacta and the left nucleus accumbens core in some rats, and the left subtantia nigra pars compacta and right nucleus accumbens core in other rats. The model also predicts that a lesion of the dorsomedial striatum would slow learning more than what done by a lesion of either the prelimbic cortex or the nucleus accumbens; and that the lesion of either the prelimbic cortex or the nucleus accumbens (which form a closely integrated system for goal selection) would slower learning to a similar extent. These represent further predictions that might be tested in future empirical experiments.

The model has some limitations that, together with the opportunity to account for other phenomena related to goal-directed behavior, call for its future development in multiple directions. First, the ventral/orbital prefrontal cortex of the model is now connected to the dorsal prefrontal/associative cortex that in turn is connected to the motor cortex. This was done as currently the model associative loop does not play any specific function while in animals it serves important functions, such as working memory and the control of attention and affordances (e.g., see Baldassarre et al., 2013b; Fiore et al., 2014, for some examples). Second, the model has now one action for each goal whereas it would be more realistic to have several possible actions for each goal. Such actions might be selected by bottom-up information from the environment reaching the premotor/motor cortex via sensory associative areas (see Chersi et al., 2013, for an example). Third, the goal representations in the prelimbic cortex are rather abstract. The prefrontal cortex receives input from multiple sources of information that allow it to encode goals in terms of multimodal, rich sets of features (Passingham and Wise, 2012). The addition to the model of afferent connections carrying such information would allow the introduction of learning processes happening within the prefrontal cortex and leading to the progressive formation of goal representations.

A further issue, moving toward the use of the model to account for different phenomena relevant for goal-directed behavior, involves contingency degradation experiments. Contingency degradation is a second experimental paradigm that, together with devaluation, has been used to establish goal-directed behaviors in mammals (Balleine and Dickinson, 1998). This paradigm manipulates the probabilities of obtaining a certain outcome (e.g., a food) if a certain action (e.g., pressing a lever) is performed or is not performed. The (action-outcome) contingency is degraded by making such probabilities similar, e.g., by delivering a food, previously obtainable by pressing a lever, even without its pressure. In the future, the model presented here could be extended to account for contingency degradation effects by adding a learning process directed to form the cortical and sub-cortical connections that link goal representations of accumbens and prelimbic cortex to action representations of the motor basal ganglia-cortical loop (e.g., see Baldassarre et al., 2013b).

Similarly, the model does not currently account for Pavlovian instrumental transfer (PIT), an important phenomenon closely related to devaluation (Corbit and Balleine, 2005; Cartoni et al., in press). PIT comes in two forms, a specific and an aspecific one. Specific PIT, more relevant for this work, is typically shown with a three-phases experimental paradigm. In a first instrumental phase, rats are trained to associate each of two different manipulanda (e.g., levers) to a different reward (e.g., two foods). In a second Pavlovian phase, rats are trained with a Pavlovian procedure to associate a cue to one of the two rewards used in the previous phase. In a third test phase, rats access each of the two manipulanda with or without the presence of the Pavlovian cue. The typical result is that when the Pavlovian cue is present rats tend to press the lever associated to the reward linked to the cue more frequently than the other lever. Specific PIT is relevant for this work because, as devaluation, it involves both Pavlovian and instrumental processes and is impaired by a lesion of the basolateral amygdala (Blundell et al., 2001; Corbit and Balleine, 2005), although, interestingly, contrary to devaluation it is impaired by the lesion of nucleus accumbens shell rather than core (Corbit et al., 2001). Accounting for specific PIT and understanding how it relates to devaluation represents an important challenge for future work (see Cartoni et al., 2013, and Bradfield et al., 2015, for two probabilistic models capturing functional aspects of PIT).

Notwithstanding the need of these future extensions, the model contributes to the elaboration of an overall theory on brain mechanisms underlying instrumental devaluation effects and in particular on how incentive value is assigned to goals in goal-directed behavior. In particular, we think the main aspects of this contribution are two. First, the integration of data on lesions involving devaluation into a coherent operational model. Second, the proposal of an overall system-level architecture that, although abstract at the level of the single components, connectivity, and learning processes, represents an important “skeleton” usable to build more detailed future theories and models of devaluation and goal-directed behavior.

## Author contributions

Idea of work: FM, MM, GB; implementation of model and simulations: FM; analysis of results: FM, MM, GB; writing up of article: FM, MM, GB.

### Conflict of interest statement

The authors declare that the research was conducted in the absence of any commercial or financial relationships that could be construed as a potential conflict of interest.

## Appendix: acronyms and model parameters

Table A1 reports the acronyms used throughout the paper. Main components of the model corresponding to some of the acronyms are also summarized in Figure 3.

**Table A1:** **Acronyms used in the paper**.

Amg	Amygdala
BG	Basal ganglia
BGdl	Basal ganglia, dorsolateral part
BGdm	Basal ganglia, dorsomedial part
BGv	Basal ganglia, ventral part
BLA	Amygdala, basolateral complex
CeA	Amygdala, central nucleus
CS	Conditioned stimulus
Ctx	Cortex
DA	Dopamine
DLS	Dorsolateral striatum
DM	Dorsomedial thalamus
DMS	Dorsomedial striatum
GPe	Globus pallidus, external part
GPi	Globus pallidus, internal part
Hip	Hippocampus
Hyp	Hypothalamus
IC	Insular cortex, gustatory division
IDE	Instrumental devaluation effects
LH	Lateral hypothalamus
MC	Motor cortex
MD	Mediodorsal thalamus
MGV	Thalamus medial geniculate body, ventral division
NAc	Nucleus accumbens, core part
NAs	Nucleus accumbens, shell part
OFC	Orbitofrontal cortex
P	Pulvinar, part of thalamus
PDE	Pavlovian devaluation effects
PFC	Prefrontal cortex
PFCd	Prefrontal cortex, dorsal division
PFCm	Prefrontal cortex, medial division
PL	Prelimbic cortex
PMC	Premotor cortex
PPC	Posterior parietal cortex
PPN	Peduncolopontine nucleus
SNpc	Substantia nigra pars compacta
SNpr	Substantia nigra pars reticulata
S, O, R, A	Stimulus, outcome, response, action
STN	Subthalamic nucleus
STNdl	Dorsolateral subtalamic nucleus
STNdm	Dorsomedial subtalamic nucleus
STNv	Ventral subtalamic nucleus
TC	Temporal cortex
Th	Thalamus
UR	Unconditioned response
US	Unconditioned stimulus
VTA	Ventral tegmental area

Tables A2–**A4** indicate the values of the parameters used in the model. These parameters were hand-tuned with a trial-and-error search aiming to find sufficient conditions (model equations and parameter values) for the reproduction of the target experiments. At this stage of the research, it was not possible to carry out an automatic research and exploration of the parameters given the long time taken to run all the lesion simulations (order of hours), in turn due to the need for time consuming learning processes. We hence set the model parameters by hand according to these criteria: (a) the values were able to reproduce the target experiments used as model constraints; (b) the parameter values were as much as possible homogeneous for different portions of the same structure (e.g., for different districts within BG or Ctx); (c) the found values had some robustness, meaning that we changed the values above and below the reported values and ensured that the model kept reproducing the results (given the aforementioned computational constraints, this sensitivity analysis was non-systematic as it was carried out only for the values of the other parameters found that far).

**Table A2:** **Model architecture: parameters of neural units**.

**Neural components**	**Parameters of neural units**				
**BG**	τ	σ	θ		
DLS	300	1	0		
STNdl	300	1	0		
GPi	300	1	0		
DMS	300	1	0		
STNdm	300	1	0		
GPi/SNpr	300	1	0		
NAc	300	1	0		
STNv	300	1	0		
SNpr	300	1	0		
**Th**	τ	σ	θ		
MGV	300	1	0		
P	300	1	0		
DM	300	1	0		
**Ctx**	τ	σ	θ		
MC	2000	20	0.8		
PFCd/PC	2000	20	0.8		
PL	2000	20	0.8		
**DA**	τ	σ	θ		
SNpco	300	1	1		
SNpci	300	1	1		
VTA	300	1	1		
**Onset units**		σ	θ	τ_*o*_	τ_*i*_
PPN		1	0	100	500
LH		1	0	100	500
BLA		1	0	500	500

Table A2 indicates the values of the parameters of the neural units of the model. For the units of BG and Th, we did not use any special threshold (θ = 0) and tanh function pendence (σ = 1), i.e., we used the function in its default form; moreover we used a time coefficient (τ = 300 ms) that ensured a response of the units at a time scale compatible to the one of the input stimuli. The Ctx units had a slower reactivity (τ = 2000) to ensure a longer integration of information, a high threshold (θ = 0.8), and a high tanh function (σ = 20), to ensure a prompt triggering of actions only when enough information was accumulated by the BG-Th-Ctx channels (action triggering happened with an activation θ_*mc*_ > 0.8 of motor cortex units, see below). Dopaminergic units, belonging to structures often considered part of BG, have the same parameter values of the latter but a high threshold (θ = 1) to fire DA bursts only when highly excited. Onset units of PPN, LH, and BLA had standard tanh-pendence (σ = 1) and threshold (θ = 0) values. Moreover, PPN and LH had a fast responsive output (τ_*o*_ = 100), followed by a slower deactivation (τ_*i*_ = 500), to cause a sudden DA production in target DA structures. Instead, BLA units had a similar activation and deactivation (τ_*o*_ = 500 and τ_*i*_ = 500) to ensure a slow responsivity useful to detect the temporal relations of input events.

Table A3 indicates the values of the parameters of the connections between the different components of the model and within them. The connectivity strength internal to the BG (Str-GPi/SNpr and STN-GPi/SNpr connections), and between BG and Th (GPI-SNpr connections), was regulated to lead BG to perform a suitable selection of Ctx contents (Gurney et al., 2001), while also considering the important effects of the re-entrant activations from the within-loop cortical areas (Ctx-Str and Ctx-STN connections; Fiore et al., 2014). Regarding the cortical connections between different loops, the stronger connections from PFCd/PC to MC and PL in comparision to the opposite connections assigned to PFCd/PC a predominant role during learning that allowed it to form the needed links between the goal and the motor loops. Regarding DA production, the strong activation of PPN and LH toward respectively SNpc and VTA, and the strong disinhibition of the latter ones by respectively DMS and NAc, were needed to produce the strong DA transients typical of dopaminergic phasic bursts guiding learning. The food input signals to PPN and LH, and the satiation signals to BLA, were set to high values, with respect to manipulanda inputs, for their role in learning.

**Table A3:** **Model architecture: connection weights between and within the model neural components**.

**Receiving components**	**Sending components**				
**BGdl**	Mani	MC	DLS	STNdl	
DLS	L	1			
STNd		1.6			
GPi			−3	−2	
**BGdm**	Mani	PFCd/PC	DMS	STNdm	
DMS	L	1			
STNdm		1.6			
GPi/SNpr			−3	−2	
**BGv**	BLA	PL	NAc	STNv	
NAc	L	1			
STNdm		1.6			
SNpr			−3	−2	
**Th**	MGV	P	DM	GPi/SNpr	
MGV	−0.8			1.5	
P		−0.8		1.5	
DM			−0.8	1.5	
**Ctx**	MC	PFCd/PC	PL	Th	
MC		1		1	
PFCd/PC	0.2		0.2	1	
PL		1		1	
**DA**	SNpci	PPN	LH	DMS	NAc
SNpco	1	20			
SNpci				−10	−6
VTA			20		
**Onset units**	Mani	Food	Sat	BLA	
BLA	5	5	10	L	
PPN		10			
LH		10		5	

“L” indicates “learned” connections (initially set to zero). “Mani” indicates “manipulandum”. “Sat” indicates “satiation.”

Table A4 indicates the values of the parameters related to the exploration noise and the learning processes of the model. The noise parameter is higher for the goal loop as exploration is not facilitated by the inputs (as in the case of the motor and associative loops): indeed, initially the input that NAc receives from BLA involves only food but not other stimuli. Regarding BLA learning, the amplification coefficient (α) has to be very high due to the signal filters of the onset units (Equation 4) and the memory trace (Equation 6) that smooth the signal spikes needed to detect relevant events by BLA (Equation 7). Regarding DA based activation (ι parameters), DLS, DMS, and NAc have an increasing stimulus-independent dopamine-based activation response reflecting the increasing responsiveness of these BG districts in correspondence to a given DA baseline activation (Fiore et al., 2014). The stimulus-dependent DA effect (δ parameters) of the associative loop was set to higher values with respect to those of the motor and goal loops to allow it to suitably “bridge” the other two loops. Regarding striatal learning, we found the model needed a higher selectivity before triggering learning of the goal-loop (θ_*da, NAc*_ and θ_*inp, NAc*_) with respect to the other loops to ensure the formation of focused links between goals and associative/action representations. This was compensated with a higher learning rate (η_*NAc*_). The higher maximum value of the BLA-NAc connection weights (*max*_*w, NAc*_) with respect to those of the other striatal regions ensured a stronger control of internal values (BLA) on goals (NAc) with respect to stimuli on actions and associations (DLS, DMS). Finally, regarding MC an action was triggered when its encoding unit overcome a threshold θ_*MC*_ = 0.8.

**Table A4:** **Noise, learning, and dopamine-related parameters used in the model**.

**Noise thalamus**		
τ	80	Noise decay constant
ν_*MGV*_	0.25	Noise coefficient of MGV
ν_*P*_	0.25	Noise coefficient of P
ν_*DM*_	6.0	Noise coefficient of DM
**BLA learning**		
τ_*tra*_	500	Trace time constant
α	10^10^	Trace amplification coefficient
η_*bla*_	0.08	BLA/IC learning rate
θ_*da, bla*_	0.7	BLA/IC DA learning threshold
*max*_*w, bla*_	2	Maximum connection weight in BLA/IC
**Str DA-based activation**		
ι_*DLS*_	0.2	DLS dopamine independent input coefficient
ι_*DMS*_	0.5	DMS dopamine independent input coefficient
ι_*NAc*_	0.8	NAc dopamine independent input coefficient
δ_*DLS*_	4.0	DLS dopamine dependent input coefficient
δ_*DMS*_	6.5	DMS dopamine dependent input coefficient
δ_*NAc*_	1.5	NAc dopamine dependent input coefficient
**Str learning**		
η_*DLS*_	0.02	DLS learning rate
η_*DMS*_	0.02	DMS learning rate
η_*NAc*_	0.05	NAc learning rate
θ_*da, DLS*_	0.8	DLS dopamine learning threshold
θ_*da, DMS*_	0.8	DMS dopamine learning threshold
θ_*da, NAc*_	0.9	NAc dopamine learning threshold
θ_*DLS*_	0.5	DLS activation learning threshold
θ_*DMS*_	0.5	DMS activation learning threshold
θ_*NAc*_	0.9	NAc activation learning threshold
θ_*inp, DLS*_	0.5	DLS input activation learning threshold
θ_*inp, DMS*_	0.5	DMS input activation learning threshold
θ_*inp, NAc*_	0.9	NAc input activation learning threshold
*max*_*w, DLS*_	1	Input-DLS maximum connection weight
*max*_*w, DMS*_	1	Input-DMS maximum connection weight
*max*_*w, NAc*_	2	BLA-NAc maximum connection weight
**MC**		
θ_*mc*_	0.8	Threshold for triggering actions
